# Prostate cancer risk biomarkers from large cohort and prospective metabolomics studies: A systematic review

**DOI:** 10.1016/j.tranon.2024.102196

**Published:** 2024-11-23

**Authors:** Yamilé López-Hernández, Cristina Andres-Lacueva, David S. Wishart, Claudia Torres-Calzada, Miriam Martínez-Huélamo, Enrique Almanza-Aguilera, Raul Zamora-Ros

**Affiliations:** a Department of Biological Sciences, University of Alberta, Edmonton, Alberta, Canada, T6G 2E9; b Biomarkers and Nutrimetabolomics Laboratory, Department of Nutrition, Food Science and Gastronomy, Research Institute of Nutrition and Food Safety (INSA-UB), Faculty of Pharmacy and Food Science, University of Barcelona (UB), 08028, Barcelona, Spain; c CIBER of Frailty and Healthy Aging (CIBERFES), Instituto de Salud Carlos III, 28029, Madrid, Spain; d Unit of Nutrition and Cancer, Cancer Epidemiology Research Program, Catalan Institute of Oncology (ICO), Bellvitge Biomedical Research Institute (IDIBELL), 08908, Barcelona, Spain

**Keywords:** Prostate cancer, Metabolites, Cohort studies, Nutrition, Biomarkers

## Abstract

Prostate cancer (PCa) is one of the leading causes of cancer-related deaths among men. The heterogeneous nature of this disease presents challenges in its diagnosis, prognosis, and treatment. Numerous potential predictive, diagnostic, prognostic, and risk assessment biomarkers have been proposed through various population studies. However, to date, no metabolite biomarker has been approved or validated for the diagnosis, prognosis, or risk assessment of PCa. Recognizing that systematic reviews of case reports or heterogenous studies cannot reliably establish causality, this review analyzed 29 large prospective metabolomics studies that utilized harmonized criteria for patient selection, consistent methodologies for blood sample collection and storage, data analysis, and that are available in public repositories. By focusing on these large prospective studies, we identified 42 metabolites that were consistently replicated by different authors and across cohort studies. These metabolites have the potential to serve as PCa risk-assessment or predictive biomarkers. A discussion on their associations with dietary sources or dietary patterns is also provided. Further detailed exploration of the relationship with diet, supplement intake, nutrition patterns, contaminants, lifestyle factors, and pre-existing comorbidities that may predispose individuals to PCa is warranted for future research and validation.

## Highlights

• To the best of our knowledge, this is the first systematic review exclusively focused on plasma/serum risk assessment biomarkers for prostate cancer (PCa), selected through a careful examination of large prospective studies. By focusing only in homogeneous studies and similar inclusion criteria and methodologies, we can provide more conclusive results.
• We propose a preliminary set of 42 metabolites potentially involved in PCa development and progression. Those metabolites were coincident across at least two large cohort studies included in the present work, where sample collection was done years before PCa diagnosis. Some of these metabolites, such as citrate, may be considered for translational clinical applications.
• Correlations between metabolites and dietary sources are evident for few of the detected metabolites, although further investigation is needed to establish the associations between both dietary and environmental exposures, and PCa risk and prognosis.

## Introduction

In 2020, prostate cancer (PCa) was the second most commonly diagnosed malignancy worldwide, following lung cancer, and the third leading cause of cancer-related deaths among men. With 1,414,259 new cases and 375,304 deaths reported globally, PCa represents a major health concern for men worldwide [1]. When analyzed by region, Northern and Western Europe, the Caribbean, Australia/New Zealand, North America, and Southern Africa have the highest incidence rates, while Asia and North Africa report the lowest incidence. The incidence of PCa rose in the 1980s and 1990s primarily due to the introduction of the Prostate-Specific Antigen (PSA) test, which enabled the detection of preclinical cancers [1]. However, PSA-based screening for PCa may not only lead to overdiagnosis and overtreatment, but also fail to detect cancer in approximately 15 % of men screened when levels are ≤ 4 ng/mL [2].

PCa is a complex disease with considerable variability in its clinical presentation, behavior, and molecular characteristics. Its management and outcomes can be influeneced by factors such as age, overall health, ethnicity, genetics, family history, comorbidities (including obesity, infections, and inflammation), environmental exposures, diet, and ionizing radiation exposure [4]. This heterogeneity poses challenges for diagnosis, prognosis, treatment selection, and patient management. Therefore, to overcome these complexitites, there is an urgent need for improved diagnostic approaches.

Numerous studies have prospectively evaluated the impact of dietary habits, lifestyle, and nutrition on the development of PCa. For example, the traditional Mediterranean diet has shown a weak or minimal association with overall PCa risk [3,4], while a proinflammatory diet has been identified as a risk factor for PCa development [5]. A potential direct association has been observed between the consumption of red meat, processed meat, and total meat [6,7] and the risk of PCa, while no associations have been found for fish consumption or intake of fish-derived omega-3 fatty acid [7,8]. Foods rich in lycopene and isoflavones, such as daidzein, genistein, and glycitein have shown some protective effects against PCa [[9], [10], [11]]. However, most of these dietary studies relied on less reliable diet assessment tools, such as self-reported or memory-based dietary questionnaires (i.e., 24 h dietary recalls or food frequency questionnaires).

While a number of reasonably reliable diagnostic protein and mRNA biomarkers for PCa are known and/or have been approved for clinical use [12], there are no approved pre-diagnostic (or predictive) PCa biomarkers and only a modest number of proposed PCa metabolite biomarkers for diagnosis, prognosis or prediction exist. Metabolomics, with its high sensitivity and precision, is a powerful tool that enables the elucidation of the metabolic phenotype associated with a disease. Two systematic reviews [13,14] have been published to summarize the various PCa metabolite findings identified over the years in different biofluids or tissue samples using metabolomics approaches (Supplementary Table 1). These reviews analyzed different types of proposed biomarkers, including risk assessment, diagnostic, and prognostic biomarkers found through various analytical strategies and experimental designs. Major limitations reported in these studies include small sample sizes and lack of validation. For instance, the systematic review conducted by Kdadra et al. included 17 blood-based metabolomic studies, of which only six were nested case-control studies focused on PCa risk [[15], [16], [17], [18], [19], [20]]. Similarly, the systematic review by Bansal et al. analyzed 27 blood-based metabolomic studies, with only eight involving large cohorts for PCa risk assessment [[15], [16], [17], [18], [19], [20], [21], [22]].

Our manuscript seeks to address some of the limitations highlighted by Kdadra et al. and Bansal et al. in their previously published systematic reviews [13,14]. After reviewing 59 studies that analyzed blood, urine, and tissue samples to identify biomarkers for PCa diagnosis, prognosis, progression, aggressiveness, recurrence, and treatment response, Kdadra et al. emphasized the need for multicenter studies with large sample sizes and consistent pre- and post-analytical methods. Similarly, Bansal et. al., in their review of 27 studies focused on serum/plasma metabolite biomarkers for PCa identification, progression, or recurrence, noted limitations in sample size—ranging from several dozen to up to 100 cases per group—, sample diversity across different continents, and variability in analytical platforms. The studies included varied widely in experimental design, types of biological fluids used, and analytical techniques employed. The experimental designs, whether it be a cohort study, nested case-control study, or cross-sectional study, leads to the discovery of different types of biomarkers, either diagnostic, prognostic, predictive, or for risk assessment. This heterogeneity makes it difficult, if not impossible, to propose robust and specific biomarkers for validation. Our study aims to address this by specifically including prospective large cohort studies with harmonized criteria for sample selection, study design, specimen types, and comparable follow-up, which distinguishes our work from previous studies.

Large cohort and multicentric studies with harmonized inclusion criteria, well-established methods for sample collection and storage, rigorous data collection, validated questionaries, and extensive follow-up are crucial for identifying significant biomarkers for any disease, including PCa. Moreover, prospective studies not only provide important data for identifying disease risks but also offer evidence on how diseases develop and can be detected earlier, thereby improving the chances of successful treatment and prolonged survival. In this review, we summarize the PCa pre-diagnostic and PCa risk-assessment metabolites found to be altered in a number of large prospective studies involving incident PCa cases. These cases have been followed up for at least one year from the time of sample collection to diagnosis. Although we acknowledge the value of different study designs, by excluding smaller sample sizes and studies based on other biological fluids, we aimed to improve the interpretability of results, particularly regarding the metabolites associated with PCa reported in multiple large cohort study.

## Materials and methods

### Search strategy

For the purposes of identifying PCa risk-assessment biomarkers, we searched for large prospective cohort studies that included men who were diagnosed with PCa ten years (as average) after sample collection. From October to November 2023, we first screened different publicly repositories, such as the Pooling Project of Prospective Studies of Diet and Cancer (DCPP) (https://www.hsph.harvard.edu/pooling-project/), the COnsortium of METabolomics Studies (COMETS) database (https://cssi.cancer.gov/comets), the web-based Cancer Epidemiology Descriptive Cohort Database (CEDCD) (https://cedcd.nci.nih.gov), and Cohorts Developed by Division of Cancer Epidemiology and Genetics (DCEG) Investigators (https://dceg.cancer.gov/research/who-we-study/cohorts).

The DCPP is an international consortium of 39 cohort studies, highly harmonized at the participant level with standardized criteria across studies. It is independently designed to investigate associations between dietary and anthropometric factors and cancer risk. The COMETS database includes prospective cohorts with more than 100 participants with blood metabolomics data. As of December 2021, 46 projects with identified metabolite data acquired by mass spectrometry and nuclear magnetic resonance spectroscopy have been listed in COMETS. The CEDCD aims to enhance awareness of resources, facilitate interdisciplinary research collaborations, and support existing cohorts for the study of cancer-related outcomes.

Large cohort studies collecting serum/plasma samples before cancer diagnosis were selected. We defined large cohort studies as observational research studies that follow a large group of individuals (the cohort, ranging from 10,000 to several thousands of participants) over a period of time to investigate associations between exposures (e.g., lifestyle factors, environmental exposures, genetic markers) and specific outcomes (e.g., development of diseases).

After the selection of cohorts from the public repositories, we conducted a search on PubMed (https://pubmed.ncbi.nlm.nih.gov) using the following combination of MeSH terms and Boolean operators: (“name of the cohort study” OR abbreviation) AND “prostate cancer” AND (metabolomics OR metabol*). Titles and abstracts of all identified studies were screened and reviewed based on the established selection criteria. Only articles relevant to the topic of interest were selected, without date restriction. Duplicates were excluded. We scrutinised the reference sections of retrieved publications for other reports. Two reviewers (YLH and CTC) independently screened the articles identified from the various databases according to their titles and abstracts.

### Selection criteria

Once the initial screening based on paper titles and abstracts was done, the following inclusion criteria were applied: only full-text articles in English were reviewed. Only prospective/longitudinal/observational/large cohort metabolomics studies that used serum/plasma samples collected at baseline, with a PCa diagnosis made within at least one year after sample collection as the main outcome, were included.

Reviews and studies conducted on animal models or cell model systems were excluded, as well as editorials, commentaries, letters, conference abstracts/proceedings, and meta-analyses). We adhered to criteria for systematic reviews as outlined in the PRISMA guidelines [15].

### Data extraction

The following information was retrieved from each of the selected studies: name of the first author, year of publication, sample size, study cohort name, analytical platform used, metabolomic approach (targeted, untargeted), results (absolutely quantitative, semi-quantitative, non-quantitative/relative), statistical methods, PCa outcome, and the relevant metabolite biomarker candidates. We defined untargeted studies are those in which thousands of unknown features are detected, with non-quantitative/relative intensities reported for each evaluated condition. Conversely, targeted metabolomics approaches analyze a set of metabolites linked to common chemical classes or a selected metabolic pathway. The results of these studies are reported as either quantitative analyses (absolute concentrations) or semi-quantitative (relative intensities) [16].

### Quality assessment

Study quality and risk of bias were evaluated using the Risk-of-Bias tool version 2 (RoB2) as outlined in the Cochrane Handbook for Systematic Reviews of Interventions (www.training.cochrane.org/handbook). The domains evaluated were: Bias in Identification of Participants (Domain 1a); Bias in Recruitment (Domain 1b); Bias Due to Deviations from Intended Interventions (Domain 2); Bias Due to Missing Data (Domain 3); Bias in Measurement of Outcomes (Domain 4); Bias in Selection of Reported Results (Domain 5).

The Risk-of-Bias assessments of each study were conducted independently by two authors (YLH and CTC).

## Results

### Cohort studies screening

**Fig. 1:**
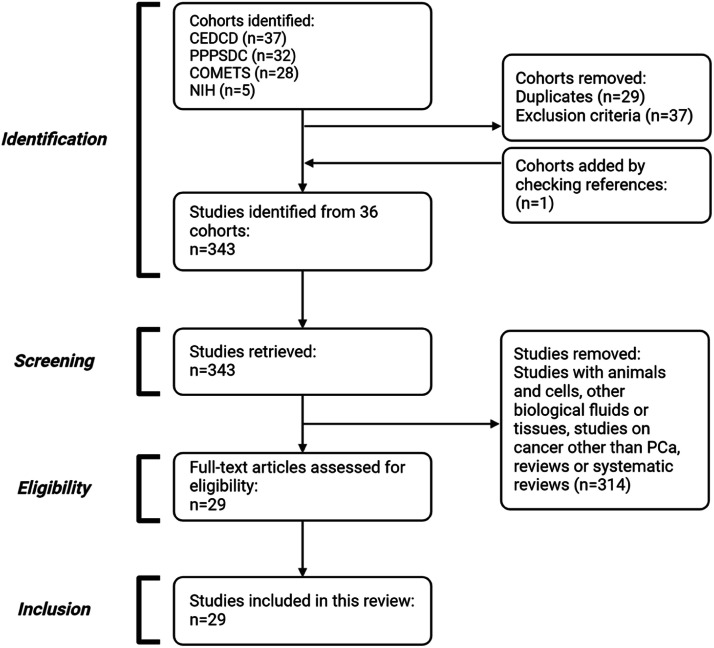
Flow chart based on PRISMA guidelines [15].

After screening the CEDCD, PPPSDC, COMETS and cohorts developed by the DCEG investigators, 102 cohort studies were identified (Fig. 1). Of those, 29 cohort studies were eliminated as they were duplicated across all repositories. An additional 37 cohort studies were removed as they did not meet the inclusion criteria (studies were either conducted on women, children, or with purposes other than cancer evaluation). One cohort was added through reference identification.

**Fig. 2:**
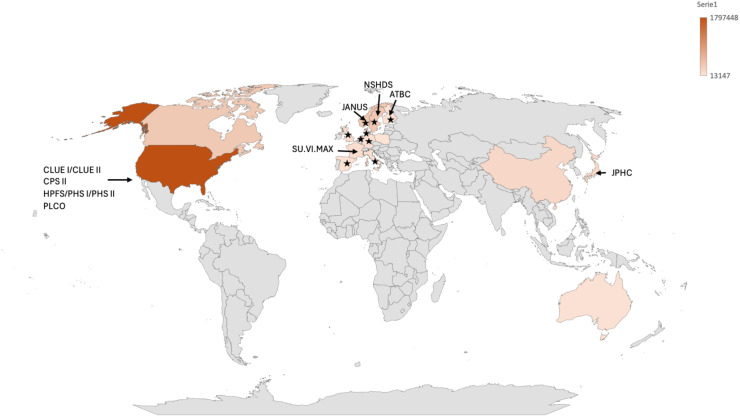
Map representing the countries participating in 13 screened cohort studies. The map shows cohort studies with PCa patients. Serie 1 represents the number of participants in PCa studies. Countries participating in the EPIC study are representd by *.

Supplementary Table 2 shows the final set of selected cohorts. In total, PCa studies were conducted in 17 countries (Fig. 2), including 3,748,152 participants. Based on the available data, nearly 129,000 incident PCa cases have been documented across the included studies.

### PCa studies selected from large cohorts

When the 37 cohort studies were screened in PubMed, metabolomics analyses were reported for 13 cohorts which were published in 29 separate papers.

Table 1 shows the 29 studies conducted with patients recruited from 13 cohorts. Ten studies used fully quantitative targeted metabolomics approaches, while 13 employed untargeted methods (semi-quantitative). Six studies used gas chromatography or liquid chromatography methods with detectors other than mass spectrometers. These non-MS-based studies were also labeled as quantitative (two studies) or non-quantitative/relative (four studies). Within the ATBC cohort (Finland), six studies were conducted, all of which were untargeted or semi-quantitative targeted studies with Metabolon (Durham, NC, USA) as the primary analytical platform. Within the EPIC cohort (Europe), four studies were conducted using targeted, fully quantitative metabolomic approaches (Biocrates, Innsbruck, Austria) and two semi-quantitative studies using gas chromatography with other methods for mass detection. Within the HPFS cohort, four studies were conducted: two untargeted studies and semi-quantitative studies using gas chromatography. There was no significant variation in the statistical analyses described in the reported studies, with logistic regression and Cox proportional hazards regression analyses being predominant, employing different version of SAS (SAS Institute, Cary, NC, USA), STATA (StataCorp LP,College Station, TX) or R (R Development Core Team, Vienna, Austria).

**Table 1:** Studies included from each cohort selected for systematic review

Cohort	Author	Type of study	Time between sample collection and diagnosis (years)	Participants	Type of analysis	Methodology	Statistics
JANUS	de Vogel et al. [17]	NCC	15.6	3000 PCa; 3000 controls	Targeted metabolomics, quantitative	LC/MS and GC/MS	conditional logistic regression (SAS 9.2 software and R 2.14.1 )
	Koutros *et al.*[25]	NCC	17.8	150 PCa; 314 controls	Targeted metabolomics, quantitative	GC-HRMS	conditional logistic regression. Sensitivity analysis for reverse causality (SAS 9.2 software)
	Harvei *et al.*[26]	NCC	11.6	141 PCa; 282 controls	Quantitative	GC/LC	conditional logistic regression
CLUE I/CLUE II	Huang et al. [27]	NCC	17 /3.5	182 PCa/364 controls 142 PCa/284 controls	Quantitative	LC with multiwavelength detection	conditional logistic regression
CPS-II Nutrition	Wang et al. [28]	NCC	10	241 PCa; 347 controls	Untargeted metabolomics, semi-quantitative	RP/UPLC-MS/MS	Cox proportional hazard regression models
HPFS/PHS I/ PHS II	Feng et al. [29]	NCC	5.5	HPFS: 294 controls; 277 PCa with ERG status and 211 with PTEN status. PHS: 181 PCa; 100 controls.	Untargeted metabolomics, semi-quantitative	LC-MS	multinomial logistic regression. sensitivity analysis for reverse causality (SAS 9.4 software and R 3.6.1)
	Dickerman et al. [30]	NCC	5.5	HPFS: 212 advanced PCa (T3b/ T4/ N1/ M1 or lethal;212 controls	Untargeted metabolomics, semi-quantitative	LC-MS	logistic regression. sensitivity analysis for reverse causality
	Yang et al. [31]	NCC	9	PHS: 476 PCa; 476 controls	Semi-quantitative	GC	conditional logistic regression (SAS version 9.3)
	Chavarro et al. [32]	NCC	9	PHS: 476 PCa; 476 controls	Semi-quantitative	GC	conditional logistic regression
ATBC	Mondul et al. [19]	NCC	1 to 23	74 PCa;74 controls	Untargeted metabolomics, semi-quantitative	LC-MS and GC-MS	unmatched logistic regression (SAS version 9.1.3)
	Mondul et al. [20]	NCC	20	200 PCa (100 aggressive); 200 controls	Untargeted metabolomics, semi-quantitative	LC-MS and GC-MS	conditional logistic regression (SAS version 9.1.3)
	Huang et al. [18]	NCC	10	T2 (*n* = 71), T3 (*n* = 51), or T4 (*n* = 15); 200 controls	Untargeted metabolomics, semi-quantitative	LC-MS and GC-MS	logistic regression model (SAS version 9.4)
	Huang et al. [33]	NCC	18	523 PCa; 523 controls	Untargeted metabolomics, semi-quantitative	LC-MS/MS	conditional logistic regression (SAS version 9.4)
	Huang et al. [24]	Cohort	10	197 Pca; 168 deaths	Untargeted metabolomics, semi-quantitative	LC-MS and GC-MS	Cox proportional hazards regression models (SAS version 9.4 and R version 3.6.1
	Huang et al. [34]	Cohort	11	1812 Pca; 472 PCa deaths	Untargeted metabolomics, semi-quantitative	LC-MS and GC-MS	Cox proportional hazards regression (SAS version 9.4 and R version 3.6.1
NSHDS	Östman et al. [35]	NCC	5	752 PCa; 752 controls	Untargeted metabolomics, semi-quantitative	LC-MS	conditional logistic regression
	Röhnisch et al. [36]	NCC	10.3	777 PCa; 777 controls	Targeted metabolomics, absolute quantification	AbsoluteIDQ p180 assay. NMR.	conditional logistic regression (SAS (version 9.3)
EPIC	Breeur et al. [37]	NCC	8.4	533 PCa; 533 controls	Targeted metabolomics, absolute quantification	LC-MS and FIA (AbsoluteIDQ p150)	conditional logistic regression
	Schmidt et al. [22]	NCC	9.4	3,057 matched case–CTRL	Targeted metabolomics, absolute quantification	LC-MS and FIA (AbsoluteIDQ® p180 Kit)	Treelet transform; conditional logistic regression; sensitivity analysis for reverse causality (Stata Statistical Software Package,version 15)
	Schmidt et al. [23]	NCC	9.4	1077 PCa; 1077 controls	Targeted metabolomics, absolute quantification	LC-MS and FIA (AbsoluteIDQ p180 Kit)	conditional logistic regression (Stata Statistical Software Package, version 14)
	Kuhn et al. [38]	case-cohort	6.8	774 subcohort; 310 PCa	Targeted metabolomics, absolute quantification	MetaDisIDQ™ Kit (Biocrates, Innsbruck, Austria). FIA MS/MS; LC-MS/MS	Cox proportional hazards regression ( SAS 9.3)
	Crowe et al. [39]	nested NCC	4.2	962 PCa; 1061 controls	Semi-quantitative	GC-FID	conditional logistic regression (Stata Statistical Software: Release 9)
	Dahm et al. [40]	NCC	4.2	962 PCa; 1061 controls	Semi-quantitative	GC-FID	conditional logistic regression according to quintile of TT factor scores (Stata Statistical Software: Release 9)
PLCO	Reichard et al. [41]	NCC	11.7	173 lethal PCa; 519 controls	Targeted metabolomics, absolute quantification	LC-MS/MS	Multivariable conditional logistic regression analysis (R(version 3.6.3))
	Huang et al. [21]	NCC	10	380 PCa; 380 controls	Untargeted metabolomics, semi-quantitative	UPLC-MS and GC-MS	Conditional logistic regression and PCA (SAS software version 9.3)
	Koutros et al. [42]	NCC	3.4	1122 PCa (813 non-aggressive and 309 aggressive);1112 controls	Targeted metabolomics, absolute quantification	LC-MS	Unconditional logistic regression and C-statistics
JPHC	Kurahashi et al. [43]	NCC	12.8	201 PCa; 402 controls	Targeted metabolomics, absolute quantification	LC-MS/MS	Conditional logistic regression (SAS software version 9.1)
SU.VI.MAX	Lécuyer et al. [44]	NCC	13	171 PCa; 171 controls	Untargeted metabolomics, semi-quantitative	NMR	multivariable conditional logistic regression models. To prevent reverse causality bias, sensitivity analyses were performed by excluding cases diagnosed during the first year of follow-up or with a prostatic dysfunction at baseline
	Lin et al. [45]	NCC	13	146 PCa; 272 controls	Untargeted metabolomics, semi-quantitative	UHPLC-HRMS	binomial logistic regression (R studio)

Abbreviation: FIA flow injection analysis; FID flame ionization detector; GC gas chromatography, HRMS high resolution mass spectrometry; LC liquid chromatography; MS mass spectrometry; NCC nested case-control; NMR nuclear magnetic resonance; PCa prostate cancer; UHPLC ultra high-performance liquid chromatography

The quality assessment revealed that most of the 29 studies were classified as having “low” and "moderate" risk across multiple domains. Supplementary Table 3 shows the quality assessment for each article included in this systematic review. The 29 articles demonstrated generally low risk in participant identification, recruitment, interventions, and outcome measurements, but moderate risk was common due to missing data handling and selective reporting of significant results.

### Metabolomics findings from prospective studies

A total of 237 metabolites were found to be significantly associated (either positively or negatively) with four different PCa outcomes (overall, fatal, aggressive, and non-aggressive) and mortality risk across the studies. Metabolites for which odds ratios [ORs] (95 % CI, *P* < 0.05) were reported were included in this research. Among these, metabolites reported more than once within the same cohort or in a different cohort are shown in Table 2. Of the 42 reported metabolites listed, 17 (40 %) were mentioned within the same cohort study by different authors, and 25 (59.5 %) were mentioned in different cohort studies. Nine metabolites (21 %) (citrate, glycine, glutamate, gamma glutamylphenyalanine, sphinganine, palmitic acid, myristic acid, linolenic acid, and palmitoleic acid) were reported by three studies. Thirty-seven metabolites were found to be significant in 11 untargeted (semi-quantitative) studies, while 10 metabolites were found to be significant in 8 targeted (fully quantitative) studies. C18:1, C18:2, PC ae C30:0 (lecithin) and lysoPC a C18:0 were found to be significant only in the targeted (fully quantitative) studies. Five metabolites (glycine, glutamate, choline, glutamine, and PC aa 36:2) were replicated by both targeted and untargeted approaches.

**Table 2:** Metabolites reported in at least two different cohort studies.

Metabolite	HMDB code	Metabolic Pathway/Exogenous source	Study	Years from sample collection to diagnosis	Cohort	PCa subtype/Outcome	OR (95 % CI) P-value
Citrate	HMDB0000094	Endogenous. Energy, TCA cycle/citrus fruits.	Huang et al. [21]	10	PLCO	Overall	1.54 (1.04–2.28) *P* = 0.0294
			Huang et al. [18]	10	ATBC	T4 patients	0.56 (0.32–0.99) *P* = 0.045
			Mondul et al. [20]	20	ATBC	Aggresive	0.69 (0.502–0.95) *P* = 0.0220
Glycine	HMDB0000123	Endogenous. Amino acid metabolism/plant foods in general; vegan diet.	de vogel et al. [17]	15.6	JANUS	Overall	0.83 (0.70–1.00) *P* = 0.07
			Huang et al. [34]	11	ATBC	Mortality	1.26 (1.15–1.38) *P* = 3.3 × 10^−6^
			Röhnisch et al. [36]	10.3	NSHDS	Overall	2.11 (1.21–3.69) *P* = 0.0084
Glutamate	HMDB0000148	Endogenous. Glutamate metabolism/coffee, sugar-rich diet.	Huang et al. [34]	11	ATBC	Mortality	1.36 (1.26–1.47) *P* = 6.7 × 10^−10^
			Schmith et al. [22]	9.4	EPIC	High grade	1.41 (1.02–1.97) *P* = 0.04)
			Lin et al. [45]	13	SU.VI.MAX	Overall	0.19 (0.1–0.32) *P* = 2.77 × 10^−9^
Palmitic acid	HMDB0000220	Endogenous. Fatty acid metabolism/palm oil, butter, meat, milk, and cheese.	Östman et al. [35]	5	NSHDS	Overall and non-aggressive	1.39 (1.12–1.73) *P* = 0.003; 1.37 (1.07–1.74) *P* = 0.011
			Crowe et al. [39]	4.2	EPIC	Overall	1.47 (0.97–2.23) *P* = 0.032
			Harvei et al. [26]	11.6	JANUS	Overall	2.3 (1.1–4.7) *P* = 0.02
Sphinganine	HMDB0000269	Endogenous. Sphingolipid synthesis/ Mexican oregano, jostaberries, winter squash, angelica, and epazotes.	Huang et al. [34]	11	ATBC	Mortality	1.24 (1.15–1.35) 1.9 × 10^−5^
			Lin et al. [45]	13	SU.VI.MAX	Overall	0.17 (0.10–0.26) *P* = 1.95 × 10^–13^
			Mondul et al. [19]	1–23	ATBC	Overall and aggressive cancer	0.63 (0.44–0.90) *P* = 0.0117; 0.61 (0.41–0.92) *P* = 0.0182
Gamma-glutamylphenylalanine	HMDB0000594	Endogenous. Peptide/plant-based diet.	Huang et al. [21]	10	PLCO	Overall, aggressive and non-aggressive	0.51 (0.34–0.78) *P* = 0.0015;0.57 (0.36–0.90) *P* = 0.0152; 0.65 (0.44–0.96) *P* = 0.0315
			Huang et al. [33]	18	ATBC	Lethal	1.20 (1.05–1.36) *P* = 0.007
			Huang et al. [34]	11	ATBC	Mortality	1.32 (1.23–1.42) *P* = 8.7 × 10^−8^
Myristic Acid	HMDB0000806	Endogenous. Fatty acid metabolism/most animal and vegetable fats, particularly butterfat, as well as coconut, palm, and nutmeg oils.	Mondul et al. [19]	1–23	ATBC	Overall and non-aggressive	0.60 (0.41–0.87) *P* = 0.0073;0.44 (0.23–0.84) *P* = 0.0127
			Crowe et al. [39]	4.2	EPIC	High Grade	1.64 (0.81–3.31) *P* = 0.015
			Östman et al. [35]	5	NSHDS	Overall	1.31 (1.09–1.57) *P* = 0.003
Linolenic acid	HMDB0001388	Endogenous. Fatty acid metabolism/vegan diet, margarine, whole grains.	Mondul et al. [19]	1–23	ATBC	non-aggressive	0.50 (0.27–0.94) *P* = 0.0307
			Crowe et al. [39]	4.2	EPIC	high-grade	1.79 (0.91, 3.53) *P* = 0.014
			Harvei et al. [26]	11.6	JANUS	Overall	2.0(1.1–3.6) *P* = 0.03
Palmitoleic acid	HMDB0003229	Endogenous. Fatty acid metabolism/macadamia oil (*Macadamia integrifolia*) sea buckthorn oil (*Hippophae rhamnoides*).	Mondul et al. [19]	1–23	ATBC	overall and non aggressive	0.60 (0.41–0.87) *P* = 0.0069;0.40 (0.20–0.77) *P* = 0.0062
			Chavarro et al. [32]	9	PHS	T1/T2	1.82 (1.12–2.95) *P* = 0.01
			Harvei et al. [26]	11.6	JANUS	Overall	2.8 (1.5–5.1) *P* = 0.01
2′-deoxyuridine	HMDB0000012	Endogenous. Pyrimidine metabolism/lichee, highbush blueberries, agaves, macadamia nut (*M. tetraphylla*), and red bell peppers.	Mondul et al. [20]	20	ATBC	Overall and non-aggressive	1.42 (1.092–1.85) *P* = 0.0084; 1.81 (1.152–2.85) *P* = 0.0105
			Huang et al. [21]	10	PLCO	Overall and aggressive cancer	1.47 (1.07–2.03) *P* = 0.0191; 1.50 (1.05–2.15) *P* = 0.0267
Choline	HMDB0000097	Endogenous. Phospholipid metabolism/milk, eggs, liver, and peanuts.	Huang et al. [34]	11	ATBC	Mortality	1.34 (1.24–1.43) *P* = 7.2 × 10−11
			Reichard et al. [41]	11.7	PLCO	Lethal	2.19 (1.23–3.90) *P* = 0.005
Tyrosine	HMDB0000158	Endogenous. Amino acid metabolism/sugar-rich diet.	Lécuyer et al. [44]	13	SU.VI.MAX	Overall	1.40 (1.06–1.85) P = 0.02
			Östman et al. [35]	5	NSHDS	Overall	1.68 (1.26–2.25) *P* = 0.001
Phenylalanine	HMDB0000159	Endogenous. Phenylalanine metabolism/meat, cottage cheese, and wheat germ, aspartame.	Huang et al. [34]	11	ATBC	Mortality	1.34 (1.23–1.46) *P* = 1.1 × 10^−7^
			Lécuyer et al. [44]	13	SU.VI.MAX	Overall	1.39 (1.08–1.79) P = 0.01
Aspartate	HMDB0000191	Endogenous. Amino acid metabolism/oysters, luncheon meats, sausage meat, wild game, sprouting seeds, oat flakes, avocado, asparagus, young sugarcane, and molasses from sugar beets.	Huang et al. [34]	11	ATBC	Mortality	1.35 (1.24–1.46) *P* = 2.5 × 10^−8^
			Wang et al. [28]	10	CPS-II	Fatal	1.52 (1.18––1.96) *P* = 1.1 × 10^–3^
Sarcosine	HMDB0000271	Endogenous. Amino acid derivatives/egg yolks, turkey, ham, vegetables, legumes.	de vogel et al. [17]	15.6	JANUS	Overall	0.86 (0.72–1.01) *P* = 0.03
			koutros et al. [42]	3.4	PLCO	Overall and non-aggressive	1.30 (1.02–1.65 *P* = 0.03;1.44 (1.11–1.88) *P* = 0.006
3-methylhistidine	HMDB0000479	Endogenous. Amino acid derivatives/meat consumption (chicken) and soy products.	Lécuyer et al. [44]	13	SU.VI.MAX	Overall	1.37 (1.05–1.80) *P* = 0.02
			Huang et al. [18]	10	ATBC	T2 patients	1.38 (1.07–1.79) *P* = 0.012
Glutamine	HMDB0000641	Endogenous. Amino acid metabolism/plant and animal proteins such as beef, pork, poultry, milk, yogurt, ricotta cheese, cottage cheese, raw spinach, raw parsley, and cabbage.	Lécuyer et al. [44]	13	SU.VI.MAX	Overall	1.3 (1.00–1.70) *P* = 0.047
			Breeur et al. [37]	8.4	EPIC	Advanced and localized	0.91(0.87–0.96)
N-acetyl citrulline	HMDB0000856	Endogenous. Amino acid derivative/watermelon.	Huang et al. [18]	10	ATBC	T2 patients	1.37 (1.03–1.82) *P* = 0.028
			Huang et al. [21]	10	PLCO	Overall	0.58 (0.41–0.84) *P* = 0.0034
5-Methylthioadenosine	HMDB0001173	Endogenous. Nucleoside/chia, black elderberries, kumquats, jew's ears, and pine nuts.	Huang et al. [21]	10	PLCO	Overall	0.64 (0.45–0.93) *P* = 0.0193
			Mondul et al. [19]	1–23	ATBC	Overall and aggressive cancer	1.64 (1.14–2.34) *P* = 0.0071;1.81 (1.20–2.72) *P* = 0.0044
Tryptophan	HMDB0013609	Endogenous. Amino acid metabolism/oats, bananas, dried prunes, milk, tuna fish, cheese, bread, chicken, turkey, peanuts, and chocolate.	Huang et al. [21]	10	PLCO	Overall and aggressive	0.72 (0.51–1.00) *P* = 0.0490; 0.61 (0.41–0.90) *P* = 0.0124
			Östman et al. [35]	5	NSHDS	Advanced	0.82 (0.69–0.98) *P* = 0.03
17-Methylstearate	HMDB0037397	Endogenous. Fatty acid metabolism/blackberry, cloves.	Huang et al. [21]	10	PLCO	Overall and aggressive	1.47 (1.03–2.11) *P* = 0.0354; 1.62 (1.07–2.44) *P* = 0.0225
			Huang et al. [18]	10	ATBC	T3	0.5 (0.29–0.88) *P* = 0.016
C18:2	HMDB0255895	Endogenous. Fatty acid oxidation/linoleic acid is abundant in safflower, sunflower and corn.	Röhnisch et al. [36]	10.3	NSHDS	Aggressive	0.51 (0.29–0.89) *P* = 0.0167
			Schmidt et al. [22]	9.4	EPIC	Aggressive	0.77 (0.63–0.94) *P* = 0.01
1-Palmitoleoyl-2-linoleoyl-GPC (16:1/18:2)	HMDB0013413	Glycerophospholipid metabolism/poultry, milk, and milk products.	Huang et al. [21]	10	PLCO	Overall and non-aggressive	0.60 (0.40–0.88) *P* = 0.0096; 0.58 (0.40–0.86) *P* = 0.0060
			Mondul et al. [19]	1–23	ATBC	Aggressive	0.74 (0.552–1.00) *P* = 0.0487
Tauro-beta-muricholate	HMDB0000932	Endogenous. Primary bile acid metabolism.	Mondul et al. [20]	20	ATBC	Overall	0.80 (0.642–1.00) *P* = 0.0481
			Huang et al. [21]	10	PLCO	Overall	0.68 (0.48–0.97) *P* = 0.0329
PC aa 36:2	HMDB0000593	Glycerophospholipid metabolism.	Schmidt et al. [23]	9.4	EPIC	Advanced	0.71(0.56–0.9) *P* = 0.004
			Östman et al. [35]	5	NSHDS	Aggressive	0.78(0.65–0.93) *P* = 0.0057
Alpha-ketoglutarate	HMDB0000208	Endogenous. Energy, TCA cycle.	Mondul et al. [19]	1–23	ATBC	Overall and aggressive cancer	0.53 (0.35–0.81) *P* = 0.003; 0.47 (0.30–0.74) *P* = 0.0010
			Mondul et al. [20]	20	ATBC	Aggressive	0.69 (0.512–0.94) *P* = 0.0201
Inositol 1 phosphate	HMDB0000213	Endogenous. Inositol phosphate metabolism and the phosphatidylinositol signaling system/various legumes, fruits and vegetables, beans, nuts, and other seedy food	Huang et al. [18]	10	ATBC	T3	0.6 (0.43–0.84) *P* = 0.003
			Mondul et al. [20]	20	ATBC	Overall and aggressive cancer	0.77 (0.622–0.97) *P* = 0.0231; 0.56 (0.392–0.81) *P* = 0.0024
4-imidazoleacetate	HMDB0002024	Endogenous. Histidine metabolism/grapefruit, garden onion (variety), black crowberry, and yellow zucchini	Huang et al. [18]	10	ATBC	T3 and T4	1.59 (1.11–2.3) *P* = 0.013; 2.52 (1.25–5.12) *P* = 0.01
			Mondul et al. [20]	20	ATBC	Overall and aggressive cancer	1.33 (1.082–1.63) *P* = 0.0074; 1.40 (1.042–1.89) *P* = 0.0272
Oxalate	HMDB0002329	Endogenous. Ascorbate and aldarate metabolism/vegetables, fruits, nuts, and grains	Huang et al. [33]	18	ATBC	lethal	0.83 (0.73–0.95) *P* = 0.0047
			Huang et al. [34]	11	ATBC	mortality	0.75 (0.69, 0.82) 3.0 × 10^−6^
N acetylarginine	HMDB0004620	Endogenous. Urea cycle; arginine and proline metabolism/poultry, apple	Huang et al. [18]	10	ATBC	T4 patients	0.54 (0.31–0.93) *P* = 0.028
			Huang et al. [21]	10	PLCO	Overall and aggressive cancer	0.69 (0.48–1.00) *P* = 0.0490; 0.64 (0.42–0.98) *P* = 0.0389
Stearoyl arachidonyl GPE	HMDB0009003	Endogenous. Glycerophospholipid metabolism/the stearic acid moiety is derived from animal fats, coco butter and sesame oil, while the arachidonic acid moiety is derived from animal fats and eggs.	Mondul et al. [20]	20	ATBC	Overall and aggressive cancer	0.76 (0.612–0.94) *P* = 0.0109; 0.69 (0.502–0.97) *P* = 0.0341
			Huang et al. [18]	10	ATBC	T2 and T3	0.73 (0.55–0.95) *P* = 0.019; 0.68 (0.49–0.94) *P* = 0.02
LysoPC a C18:0	HMDB0011128	Endogenous. Glycerophospholipid Metabolism/poultry.	Kuhn et al. [38]	6.8	EPIC	Overall	0.59 (0.34–1.04) *P* = 0.01
			Schmidt et al. [22]	9.4	EPIC	Advanced	0.77 (0.6–0.97) *P* = 0.02
Gamma-glutamylleucine	HMDB0011171	Endogenous. Dipeptide/onion-family vegetable.	Huang et al. [33]	18	ATBC	lethal	1.29 (1.12–1.46) *P* = 0.00046
			Huang *et al*.[34]	11	ATBC	mortality	1.31 (1.22–1.40) *P* = 2.4 × 10^−8^
Gamma-glutamylvaline	HMDB0011172	Endogenous. Dipeptide.	Huang et al. [33]	18	ATBC	lethal	1.30 (1.13–1.50) *P* = 0.00026
			Huang et al. [34]	11	ATBC	mortality	1.33 (1.24–1.42) *P* = 7.8 × 10^−8^
Gamma-glutamylglycine	HMDB0011667	Endogenous. Dipeptide/ poultry.	Huang et al. [33]	18	ATBC	lethal	1.28 (1.11–1.47) *P* = 0.00044
			Huang et al. [34]	11	ATBC	mortality	1.32 (1.23–1.41) *P* = 4.0 × 10^−9^
PC ae C30:0 (lecithin)	HMDB0013341	Endogenous. Glycerophospholipid metabolism/poultry. Milk and milk products.	Kuhn et al. [38]	6.8	EPIC	Overall	2.01(1.12–3.6) *P* = 0.001
			Schmidt et al. [23]	9.4	EPIC	Overall	1.16 (1.04–1.30) *P* = 0.007
Isoeugenol sulfate	HMDB0034135	Exogenous. Food component/plant. Nutmeg, clove, and cinnamon.	Mondul et al. [20]	20	ATBC	Aggressive	1.38 (1.012–1.89) *P* = 0.0410
			Huang et al. [18]	10	ATBC	T3	1.55 (1.06–2.25) *P* = 0.02
C18:1	HMDB0094687	Endogenous. Fatty acid oxidation.	Schmidt et al. [23]	9.4	EPIC	Overall	0.89 (0.79–0.99) *P* = 0.03
			Schmidt et al. [22]	9.4	EPIC	Aggressive	0.82 (0.68–0.98) *P* = 0.025
Salicyluric glucuronide	HMDB0240252	Exogenous. Xenobiotics	Huang et al. [18]	10	ATBC	T3	0.66 (0.45–0.96) *P* = 0.031
			Mondul et al. [20]	20	ATBC	Aggressive	0.65 (0.462–0.90) *P* = 0.0109
Cysteine gluthatione disulfide	HMDB0250707	Endogenous. Gluthatione metabolism.	Huang et al. [34]	11	ATBC	mortality	0.84 (0.78, 0.91) *P* = 1.4 × 10^−5^
			Huang et al. [18]	10	ATBC	T4 patients	0.59 (0.39–0.89) *P* = 0.012
Stearoyl linoleoyl GPE	HMDB0008994	Endogenous. Glycerophospholipid metabolism/the stearic acid moiety is derived from animal fats, coco butter and sesame oil, while the linoleic acid moiety is derived from seed oils.	Mondul et al. [20]	20	ATBC	Overall and aggressive	0.80 (0.662–0.98) *P* = 0.0329; 0.72 (0.532–0.97) *P* = 0.0308
			Huang et al. [18]	10	ATBC	T2 and T3	0.78 (0.55–0.96) *P* = 0.025; 0.64(0.45–0.93) *P* = 0.018
Oleoyl-linoleoyl-glycerophosphoinositol (GPI)	HMDB0009809	Endogenous. Glycerophospholipid metabolism/the stearic acid moiety is derived from animal fats, coco butter and sesame oil, while the linoleic acid moiety is derived from seed oils.	Mondul et al. [20]	20	ATBC	Overall and aggressive	0.81 (0.652–1.00) *P* = 0.0462;0.64 (0.472–0.87) *P* = 0.0042
			Huang et al. [18]	10	ATBC	T3	0.49(0.35–0.68) *P* = 0.000017

The studies that found the most significant and most frequently reported metabolites were conducted by ATBC, PLCO, and EPIC cohorts. Fatty acids, including myristic acid, linolenic acid, palmitic acid, and palmitoleic acid were investigated in various studies (NSHDS, EPIC, ATBC, JANUS) for overall cancer, non-aggressive, high-grade cancer, but the results were inconclusive for most of them. Palmitic acid was found negatively associated with PCa in three different cohort studies (NSKDS, EPIC, JANUS).

## Discussion

For many years, metabolomics has provided valuble insights into cancer development and progression, as well as the identification of metabolomics signatures, including potential diagnostic and prognostic biomarkers. Despite the initial identification of some promising biomarkers such as sarcosine [46], there is currently no conclusive study that proposes a feasible and validated metabolite biomarker for the diagnosis or prognosis of PCa. Indeed, no metabolite biomarker or biomarker panel has yet been found that could replace conventional PSA screening or auxiliary tools, such as trans-rectal ultrasound-guided biopsy followed by a histopathology-based Gleason score. However, there is some evidence suggesting that metabolites might be useful for predicting PCa risk. In particular, nutritional metabolomics may offer novel, objective biomarkers that reflect specific dietary and lifestyle exposures, potentially enhancing PCa risk prediction [47].

In this review, we examined large cohort studies conducted in at least 17 countries, with a particular focus on nutrition, supplement use, and lifestyle habits that may predispose individuals to chronic diseases, including PCa (https://www.hsph.harvard.edu/pooling-project/; https://cedcd.nci.nih.gov; https://dceg.cancer.gov/research/who-we-study/cohorts). Our primary goal was to identify pre-diagnostic PCa biomarkers, selecting studies that used blood samples collected from patients several years before a PCa diagnosis was confirmed (large cohort prospective studies). Identifying novel biomarkers for risk assessment is crucial for both effective disease prevention and optimal treatment recommendations. For biomarker research aimed at predicting future cancer risk, prospective cohort studies or large cohort studies with long follow-up periods are ideal as they provide a comprehensive understanding of how different biomarkers correlate with various health conditions.

On average, the follow-up time between sample collection and PCa diagnosis in these studies was 9.8 years. As showed in Fig. 1, these studies were primarily conducted in North America (USA and Canada), Europe, China, Japan, and Australia, but important regions like Latin America, Africa, and parts of Asia were underrepresented. This underrepresentation highlights the need to consider factors such as ethnicity and differences in nutritional habits or other patterns, which may affect the extrapolation of the results.

The 29 articles we evaluated are of high quality, with low to moderate risk of bias across most domains, making them largely suitable for inclusion in a systematic review. However, special attention should be given to the moderate risk of bias due to missing data, which might affect the reliability of some associations. Most studies demonstrated robust methodologies for participant identification and recruitment, with a low risk for bias in participant identification (Domain 1a) and recruitment (Domain 1b). Recruitment was generally well-structured, relying on established cohort studies that ensured representativeness and reduced selection bias. Regarding bias due to deviations from intended interventions (Domain 2), most studies adhered strictly to their protocols, resulting in a low risk in this domain. However, challenges were identified in bias due to missing data (Domain 3), where moderate risks were common due to the imputation of missing values, which introduced potential bias. In terms of bias in measurement of outcomes (Domain 4), although most studies employed reliable and validated techniques such as LC-MS/MS, some variability in measurement sites led to moderate risks in outcome measurement. Lastly, bias in the selection of reported results (Domain 5) frequently exhibited moderate risk, as many studies selectively emphasized significant findings, potentially overestimating the importance of certain associations. While the studies display methodological rigor in several domains, careful consideration is needed regarding the risks associated with missing data handling and selective reporting to ensure balanced interpretation of the findings.

Among the most frequently reported metabolites, citrate showed both positive and negative associations with PCa risk, depending on the study. In the PLCO cohort [21], citrate was positively associated with overall PCa risk, while in two studies of the ATB cohort, it was negatively associated with T4 and aggressive subtypes [18,20]. In the EPIC cohort, citrate measurements might have been affected by the use of citrate as an anticoagulant during simple collection, potentially masking the metabolite's true levels.

The apparent contradiction in citrate's associations with PCa risk might be explained by carefully considering the cancer status and deserves furhter attention. It has long been recognized that the normal human prostate gland uniquely accumulates and secretes high levels of citrate [48] due to its ability to retain zinc. Zinc inhibits the mitochondrial enzyme aconitase (ACO2), blocking the tricarboxylic acid (TCA) cycle at the first oxidative reaction, thereby leading to citrate accumulation [49]. As differentiation occurs, the prostate gland membrane disintegrates, resulting in the loss of the capacity to accumulate zinc ions, resulting in aconitase activation and a corresponding decrease in citrate levels (10–100 fold decrease compared with bening glands). Cancer cells utilize citrate for energy and growth, as tumour cells require a large amount of energy and nutrients to sustain their aberrant proliferation. Citrate is also crucial for producing metabolic energy and synthesizing cholesterol, fatty acids, and isoprenoids, all of which are essential for cancer progression. This mechanism has been described by some authors [[50], [51], [52]] in the last three years and has been supported by several experimental approaches. For instance, Buszewska-Forajta et al. [53] found that as PCa tumors progressed, the concentration of citrate decreased when the Gleason score and the concentration of serum citrate were considered. A previous study found that a combination of citrate with alanine, sarcosine, creatinine, and glycine could discriminate between PCa and benign prostatic hyperplasia [54]. Case-control studies [55,56] consistently report reduced citrate levels as a distinguishing feature of PCa compared to benign conditions. Intratumoural, urinary and seminal citrate levels have been shown to significantly decrease in PCa patients, irrespective of the quantification methods used [[55], [56], [57]].

Although case-control studies can identify potential associations between risk factors or biomarkers and the condition, they do not establish causality. Causality can only be determined through longitudinal studies—such as cohort studies, randomized controlled trials (RCTs), or Mendelian Randomization (MR) approaches. Based on the longitudinal studies analyzed, we conclude that or citrate alterations occur years before PCa diagnosis, with its levels negatively associated with tumor progression and aggressiveness.

The link between genetic variants and citrate levels is complex, involving genes that regulate citrate production and utilization. Currently, no single specific single nucleotide polymorphism (SNP) has been consistently linked to serum or tissue citrate levels in human studies. However, genetic variants affecting mitochondrial function, citrate transport, or related metabolic enzymes might play a role. Further research, such as targeted genome-wide association studies (GWAS) on citrate concentration specifically [LC13A5 (Solute Carrier Family 13 Member 5), SLC25A1 (Citrate Transporter), ACO2 (Aconitase 2), IDH (Isocitrate Dehydrogenase), Prostate-Specific Citrate Metabolism ZIP1 (SLC39A1), etc], could help identify specific genetic variants linked to serum or tissue citrate levels.

Kumar et al. [58], found that alanine, pyruvate, glycine (also reported in the present work reported within the JANUS, ATBC, and NSHDA cohorts), and sarcosine (also reported in the JANUS and PLCO cohorts) could accurately differentiate 90.2 % of PCa cases from healthy controls with a sensitivity of 84.4 % and a specificity of 92.9 %. Our findings indicate that most of the amino acids reported in two or more studies were positively associated with PCa, with the exception of tryptophan and glycine (and glutamine for overall PCa) which were negatively associated with PCa. Tryptophan, which must be obtained through the diet, requires a minimum daily intake of between 175 and 250 mg for adults. Given that the average daily intake ranges from 900–1000 mg for adults [59], the reported decrease in tryptophan concentrations in those at risk for PCa could be due to its enhanced catabolic breakdown. Tryptophan is metabolized into several biologically active compounds through the kynurenine pathway (KP) or serotonin pathway, under the action of indoleamine-2,3-dioxygenase (IDO) or tryptophan-2,3-dioxygenase (TDO), in response to inflammatory signals [60].

A recent comprehensive 2-sample Mendelian randomization (MR) study [61] evaluated the potential causal relationship between 913 plasma metabolites (quantified from the EPIC-Norfolk study and the INTERVAL study) and the risk of seven cancers among European-ancestry individuals. Untargeted plasma metabolomic profiling was conducted using the Metabolon HD4 platform. The authors found that N-acetyl tryptophan and cys-Gly oxidized (derivatives from tryptophan and glycine metabolism, respectively) were significantly associated with the risk of PCa using the inverse-variance weighted (IVW) approach.

The enhanced catabolism of glycine (i.e., lower levels of glycine in at-risk PCa individuals) may reflect the fact that glycine is required for the synthesis of purines, proteins, glutathione, and most importantly, sarcosine. Sarcosine is associated with the invasion, migration, and metastasis of PCa [46]. Lower levels of glutamine reflect an increased rate of catabolism of glutamine. Glutamine, via glutaminolysis, provides a source of carbon and nitrogen groups for the TCA cycle and NADPH for the synthesis of nucleotides, proteins, and lipids (needed for rapid cell division associated with cancer), as well as for immune system activation [62].

Previous reports have indicated a positive correlation between serum glutamate and the Gleason sum in PCa patients [63]. Heger et al. [64] found elevated levels of intracellular glutamate and aspartate in malignant prostatic cell lines, suggesting that oncogenic signaling pathways also may reprogram glutamine metabolism. Glutamate levels have been reported to increase in “inflammatory” diets that are rich in sugar [47]. Aspartate and glutamate, on the other hand, belong to the arginine family of amino acids (AFAAs), which also includes asparagine, glutamine, proline, and arginine [65]. The AFAAs are inter-convertible amino acids via complex metabolism in most mammals and are affected by dietary intake [65]. Aspartate can also be produced through the hydrolysis of aspartame in the gut. A recent study involving 102,865 adults from the French population-based cohort NutriNet-Santé (2009–2021) estimated dietary intakes and consumption of sweeteners through repeated 24 h dietary records. The study found that individuals who consumed higher amounts of total artificial sweeteners, including aspartame, were associated with an increased cancer risk [66].

Gamma-glutamyl amino acids are released when gamma glutamyl peptidase, involved in apoptotic and detoxification pathways [67], degrades extracellular glutathione. These amino acids are prevalent in edible legumes, onion-family, and fermented foods such as cheese and soy sauce. They have beneficial effects including antioxidation, anticancer, antinociceptive, antiplatelet, anti-atherosclerotic, detoxifying and lipid-lowering activities [68]. Despite the positive association found between gamma-glutamyl amino acids and fatal PCa and overall mortality risk [33,34], a protective role has been identified for overall, aggressive, and non-aggressive PCa [19].

Elevated sarcosine levels in urine [64] and serum [42] have been associated with PCa progression, although the results are conflicting [69,70]. Sarcosine, derived from glycine, is involved in both glycine's synthesis and degradation. Sarcosine is formed via the metabolism of nutrients such as choline. Given that high levels of choline and vitamin B2 have been associated with a higher risk of cancer [71], the bioavailability of methyl groups from choline and sarcosine could play a role in cancer. This justifies the interest in metabolites across the glycine-sarcosine pathway for PCa. Sarcosine is found in various foods included egg yolks, turkey, ham, vegetables, and legumes. As diet is a significant source of these metabolites, their presence in blood or urine before diagnosis could also be associated with dietary patterns.

Red meat and dairy products have been associated with an increased risk of advanced PCa, although these associations are still inconclusive [72,73]. Elevated levels of choline, a metabolite abundant in meat, milk, whole eggs, and poultry, have been found in PCa cells, and high blood concentrations of choline have been linked to an increased risk of PCa [70]. Confirmatory results were reported by Richman et al. [70] who prospectively examined the intake of choline, choline-containing compounds, and betaine and the risk of fatal PCa among 47,896 men in the Health Professionals Follow-Up Study. The findings showed that men in the highest quintile of choline intake had a 70 % increased risk of fatal PCa. In contrast, a meta-analysis of epidemiological studies [74] noted a significant protective association of dietary choline and betaine with PCa when stratified by study design, location, cancer type, publication year, sex, and quality score of the study. However, the authors acknowledged substantial heterogeneity and scarce data about observational studies, suggesting that these meta-analysis results are not particularly reliable.

In terms of lipid metabolism, phosphatidylcholines, lysophosphatidylcholines [75,76] fatty acids [77] sphingomyelins [75], acylcarnitine [78], and cholesterol [79] have been associated with different PCa risks. Elevated levels of intracellular lipids allow a higher rate of beta oxidation in mitochondria from PCa cells. High levels of fatty acids have been associated with fatty food intake and the risk of developing PCa, but results have been inconsistent. Reported results range from no association between total fat and risk of PCa [80]to an increased risk with higher intakes of alpha-linolenic acid (ALA), an *n*-3 fatty acid. A study conducted within (NIH)-AARP Diet and Health cohort found that the associations of fat consumption and fatty acid intake differed by PCa severity. Saturated fat, ALA and EPA intakes were related to the risk of advanced or fatal PCa, but not to non-advanced PCa.

Fatty acids are important constituents of the diet. Myristic acid is usually found in most animal and vegetable fats, particularly butter, as well as coconut, palm, and nutmeg oils.

Palmitic acid is found in palm oil, butter, meat, milk, and cheese, while linolenic is found in a vegan diet, margarine, whole grains, and palmitoleic acid in Macadamia (*Macadamia integrifolia*) oil and sea buckthorn (*Hippophae rhamnoides*) oil. The findings from case-control studies suggest that higher levels of palmitic acid, whether from dietary sources or present in circulation, are generally linked with an increased risk of prostate cancer, possibly through mechanisms involving inflammation, oxidative stress, and altered lipid metabolism [81]. Recent research shows that palmitic acid can activate various inflammatory mediators, such as NFκB and CCL2 [82]. This heightened inflammatory response can promote cancer development. Very recently, Dai *et al.*[83] utilized GWAS data for blood metabolites and urological cancers, using genetic variation data for derived from meta-analyses with a sample size of 412,592. Their two-sample MR study and subsequent meta-analysis reported the causal relationships between genetically determined metabolites and the susceptibility of four common urinary system cancers. They identified 1-palmitoyl-GPC, composed of palmitic acid, as a risk enhancer for bladder cancer, one of the four urinary tract cancer.

Stearoyl derivatives (Stearoyl arachidonyl GPE, Stearoyl linoleoyl GPE) were found negatively associated in studies from ATBC cohort. In line with this, Chen *et al*.[84] also found 1-Stearoyl-GPI (18:0) negatively associated with PCa in a MR study.

## Conclusions

Numerous studies have been conducted to identify PCa biomarkers that can detect the disease more efficiently than currently approved methods. However, once the disease has been established, even with silent clinical manifestations, identifying the molecular etiologic factors becomes a challenge. Therefore, the best approach for identifying PCa biomarkers (diagnostic and pre-diagnostic) is to analyze baseline data for individuals who initially did not have the disease and compare those who developed the condition with those who did not. It is well known that the incidence and prevalence of PCa are linked to non-modifiable risk factors such as ethnicity and family history of cancer, as well as to modifiable risk factors such as diet, exercise, and toxic exposures. Identifying pre-diagnostic metabolites that can reflect the status of modifiable factors is extremely important. Controlling these factors could lead to a reduction in the release of reactive oxygen species and chronic inflammation, as well as prevent collateral pathologies such as diabetes mellitus or metabolic syndrome, which have been previously associated with the development of PCa.

**Fig. 3:**
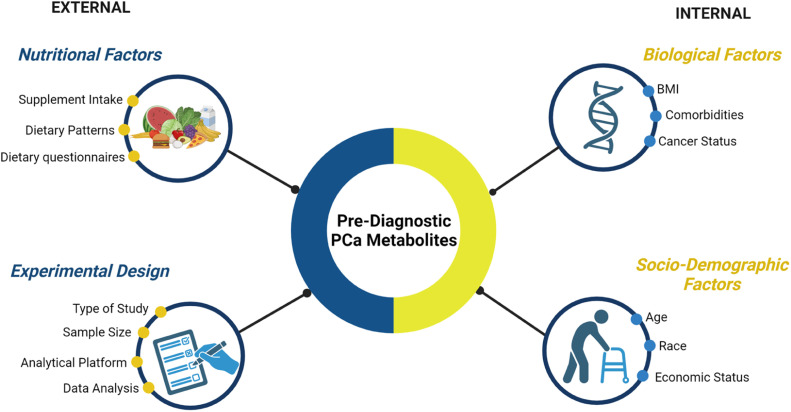
Association between biological, analytical, and external factors influencing PCa biomarkers identification.

However, the interaction between external factors (diet, supplements), biological factors (genetics, BMI, comorbidities, status of cancer at the time of diagnosis and sample collection), and sociodemographic characteristics poses challenges in finding reproducible and verifiable pre-diagnostic biomarkers. All these variables can be confounding factors or covariates and need to be adjusted in the different risk prediction models. The advantage of cohort studies is that they have validated tools for collecting all these variables in population studies. Supplementary Table 4 shows all the confounding factors or covariates that were analyzed in the 29 studies included in the systematic review, and how significant their inclusion was in the different models provided. Furthermore, different experimental designs, analytical platforms, times of sample processing, storage time, and statistical analyses can lead to divergent results (Fig. 3). Although untargeted (non-quantitative) metabolomics approaches provide the broadest metabolite coverage, the complex and lengthy data analysis can sometimes lead to misidentification, misinterpretation (of metabolite levels), or incomplete structural elucidation. Therefore, validation through targeted (quantitative) approaches is required.

In this review, we present a comprehensive summary of large cohort studies investigating PCa. Our strategy focused on an extensive review of metabolomics studies conducted in large cohorts with very similar inclusion criteria and methodology. These studies, by their very nature, have led to the identification of metabolites that appear to be dysregulated years prior the diagnosis of the disease. This early detection of metabolic changes provides a preliminary set of pre-diagnostic or predictive biomarkers. While these metabolites were identified through the analysis of large cohort studies, it is important to emphasize that the findings are still preliminary. The reliability of these findings could have been significantly enhanced if a meta-analysis had been conducted. Meta-analysis offers a more robust and precise estimate of effect sizes, enhancing both statistical power and the reliability of conclusions compared to individual studies. However, performing a meta-analysis must be approached with caution, considering two key factors: the heterogeneity among studies and the presence of reporting bias. In our case, reporting bias significantly hindered our ability to conducting a meta-analysis. A thorough analysis of all selected manuscripts, including main text, supplementary materials, and data availability statements, revealed insufficient data for certain metabolites (e.g., concentrations, odds ratios), which we recognize as a limitation of our study.

We could identify several metabolites (i.e. citrate, palmitic acid, stearoyl derivatives) that were consistently reported across different prospective cohorts and could have important translational applications. Notably, there is a significant overlap among some biomarkers mentioned across the three studies, particularly regarding amino acids, citrate, and some lipids. These biomarkers are consistently highlighted as potentially valuable in assessing the risk, progression, and prognosis of PCa, reflecting a shared focus in metabolomics-based PCa research. In the manuscript from Kdadra et al., palmitic acid was not reported. Bansal et al. mention palmitic acid (decreased) in the differentiation of PCa From Benign Prostatic Hyperplasia within the PSA Gray Zone (case-control study) [85]. Our manuscript reported more metabolites linked to nutritional sources, aligning with the nature of these cohort studies, where the main focus was to identify metabolomics biomarkers associated with lifestyles, nutrition, and their interconnection with cancer and other chronic diseases. By focusing exclusively on prospective studies, our work provides a more reliable identification of these molecules as risk assessment biomarkers.

To fully understand their association with the processes that lead to PCa, these metabolites need to be further validated. This validation should ideally be conducted in larger studies that are more representative of the global population, encompassing a diverse range of ethnic groups. Such studies will not only confirm the predictive value of these metabolites but also enhance our understanding of their role in the onset of PCa. This, in turn, could pave the way for improved diagnostic procedures and potentially more effective treatment strategies for PCa.

## Funding

The research was supported by funding contributions from La Marató de TV-3 (grants 201943-30 and 201943-31); the 10.13039/501100004587Instituto de Salud Carlos III through the project CIBERFES, CB16/10/00269, AC19/00127, and the grant PID2020-114921RB-C21 and PID2023-148013OB-C21 funded by MCIN/AEI/ all of them Co-funded by 10.13039/501100008530European Regional Development Fund, ERDF, a way to build Europe. The Generalitat de Catalunya's Agency AGAUR of 2021SGR00687. Maria de Maeztu Unit of Excellence grant (CEX2021-001234-M) funded by (MICIN/AEI/FEDER, UE).

## CRediT authorship contribution statement

**Yamilé López-Hernández:** Writing – original draft, Methodology, Investigation, Formal analysis, Data curation, Conceptualization. **Cristina Andres-Lacueva:** Supervision, Funding acquisition, Conceptualization. **David S. Wishart:** Writing – review & editing. **Claudia Torres-Calzada:** Writing – review & editing, Investigation. **Miriam Martínez-Huélamo:** Writing – review & editing. **Enrique Almanza-Aguilera:** Writing – review & editing. **Raul Zamora-Ros:** Supervision, Funding acquisition, Conceptualization.

## Declaration of competing interest

The authors declare no conflict of interest. The funders had no role in the design of the study; in the collection, analyses, or interpretation of data; in the writing of the manuscript; or in the decision to publish the results.

## Appendix Supplementary materials

Image, application 1

Image, application 2

Image, application 3

Image, application 4

## References

[1] Center M.M., Jemal A., Lortet-Tieulent J., Ward E., Ferlay J., Brawley O., Bray F. (2012). International variation in prostate cancer incidence and mortality rates. Eur. Urol..

[2] Stockert J.A., Weil R., Yadav K.K., Kyprianou N., Tewari A.K. (2021). Pseudouridine as a novel biomarker in prostate cancer. Urolog. Oncol.: Semin. Origin. Investigat..

[3] Schwingshackl L., Schwedhelm C., Galbete C., Hoffmann G. (2017). Adherence to mediterranean diet and risk of cancer: an updated systematic review and meta-analysis. Nutrients.

[4] Cheng S., Zheng Q., Ding G., Li G. (2019). Mediterranean dietary pattern and the risk of prostate cancer. Medicine.

[5] Mohseni R., Abbasi S., Mohseni F., Rahimi F., Alizadeh S. (2019). Association between dietary inflammatory index and the risk of prostate cancer: a meta-analysis. Nutr. Cancer.

[6] Nouri-Majd S., Salari-Moghaddam A., Aminianfar A., Larijani B., Esmaillzadeh A. (2022). Association between red and processed meat consumption and risk of prostate cancer: a systematic review and meta-analysis. Front. Nutr..

[7] Parra-Soto S., Ahumada D., Petermann-Rocha F., Boonpoor J., Gallegos J.L., Anderson J., Sharp L., Malcomson F.C., Livingstone K.M., Mathers J.C. (2022). Association of meat, vegetarian, pescatarian and fish-poultry diets with risk of 19 cancer sites and all cancer: findings from the UK Biobank Prospective Cohort Study and meta-analysis. BMC Med..

[8] Aucoin M., Cooley K., Knee C., Fritz H., Balneaves L.G., Breau R., Fergusson D., Skidmore B., Wong R., Seely D. (2017). Fish-derived omega-3 fatty acids and prostate cancer: a systematic review. Integr. Cancer Ther..

[9] Zhang Q., Feng H., Qluwakemi B., Wang J., Yao S., Cheng G., Xu H., Qiu H., Zhu L., Yuan M. (2017). Phytoestrogens and risk of prostate cancer: an updated meta-analysis of epidemiologic studies. Int. J. Food Sci. Nutr..

[10] Rowles J.L., Ranard K.M., Applegate C.C., Jeon S., An R., Erdman J.W. (2018). Processed and raw tomato consumption and risk of prostate cancer: a systematic review and dose–response meta-analysis. Prostat. Cancer Prostat. Dis..

[11] Rowles J.L., Ranard K.M., Smith J.W., An R., Erdman J.W. (2017). Increased dietary and circulating lycopene are associated with reduced prostate cancer risk: a systematic review and meta-analysis. Prostat. Cancer Prostat. Dis..

[12] Farha M.W., Salami S.S. (2022). Biomarkers for prostate cancer detection and risk stratification. Ther. Adv. Urol..

[13] Kdadra M., Höckner S., Leung H., Kremer W., Schiffer E. (2019). Metabolomics biomarkers of prostate cancer: a systematic review. Diagnostics.

[14] Bansal N., Kumar M., Sankhwar S.N., Gupta A. (2022). Relevance of emerging metabolomics-based biomarkers of prostate cancer: a systematic review. Expert Rev. Mol. Med..

[15] Arya S., Kaji A.H., Boermeester M.A. (2021). PRISMA reporting guidelines for meta-analyses and systematic reviews. JAMA Surg..

[16] Liu X., Locasale J.W. (2017). Metabolomics: a primer. Trend. Biochem. Sci..

[17] De Vogel S., Ulvik A., Meyer K., Ueland P.M., Nygård O., Vollset S.E., Tell G.S., Gregory J.F., Tretli S., Bjørge T. (2014). Sarcosine and other metabolites along the choline oxidation pathway in relation to prostate cancer - a large nested case-control study within the JANUS Cohort in Norway. Int. J. Cancer.

[18] Huang J., Mondul A.M., Weinstein S.J., Karoly E.D., Sampson J.N., Albanes D. (2017).

[19] Mondul A.M., Moore S.C., Weinstein S.J., Männistö S., Sampson J.N., Albanes D. (2014). 1-stearoylglycerol is associated with risk of prostate cancer: results from a serum metabolomic profiling analysis. Metabolomics.

[20] Mondul A.M., Moore S.C., Weinstein S.J., Karoly E.D., Sampson J.N., Albanes D. (2015). Metabolomic analysis of prostate cancer risk in a prospective cohort: the alpha-tocolpherol, beta-carotene cancer prevention (ATBC) study. Int. J. Cancer.

[21] Huang J., Mondul A.M., Weinstein S.J., Koutros S., Derkach A., Karoly E., Sampson J.N., Moore S.C., Berndt S.I., Albanes D. (2016). Serum metabolomic profiling of prostate cancer risk in the prostate, lung, colorectal, and ovarian cancer screening trial. Br. J. Cancer.

[22] Schmidt J.A., Fensom G.K., Rinaldi S., Scalbert A., Appleby P.N., Achaintre D., Gicquiau A., Gunter M.J., Ferrari P., Kaaks R. (2020). Patterns in metabolite profile are associated with risk of more aggressive prostate cancer: a prospective study of 3,057 matched case–control sets from EPIC. Int. J. Cancer.

[23] Schmidt J.A., Fensom G.K., Rinaldi S., Scalbert A., Appleby P.N., Achaintre D., Gicquiau A., Gunter M.J., Ferrari P., Kaaks R. (2017). Pre-diagnostic metabolite concentrations and prostate cancer risk in 1077 cases and 1077 matched controls in the European prospective investigation into cancer and nutrition. BMC Med..

[24] Huang J., Weinstein S.J., Moore S.C., Derkach A., Hua X., Mondul A.M., Sampson J.N., Albanes D. (2019). Pre-diagnostic serum metabolomic profiling of prostate cancer survival. J. Gerontol. - Ser. A Biolog. Sci. Med. Sci..

[25] Koutros S., Langseth H., Grimsrud T.K., Barr D.B., Vermeulen R., Portengen L., Wacholder S., Beane Freeman L.E., Blair A., Hayes R.B. (2015). Prediagnostic serum organochlorine concentrations and metastatic prostate cancer: a nested case-control study in the Norwegian Janus Serum Bank Cohort. Environ. Health Perspect..

[26] Harvei S., Bjerve K.S., Tretli S., Jellum E., Robsahm T.E., Vatten L. (1997). Prediagnostic level of fatty acids in serum phospholipids: Ω-3 and Ω-6 fatty acids and the risk of prostate cancer. Int. J. Cancer.

[27] Huang H.Y., Alberg A.J., Norkus E.P., Hoffman S.C., Comstock G.W., Helzlsouer K.J. (2003). Prospective study of antioxidant micronutrients in the blood and the risk of developing prostate cancer. Am. J. Epidemiol..

[28] Wang Y., Jacobs E.J., Carter B.D., Gapstur S.M., Stevens V.L. (2021). Plasma metabolomic profiles and risk of advanced and fatal prostate cancer. Eur. Urol. Oncol.

[29] Feng X., Zhou C.K., Clish C.B., Wilson K.M., Pernar C.H., Dickerman B.A., Loda M., Finn S.P., Penney K.L., Schmidt D.R. (2021). Association of prediagnostic blood metabolomics with prostate cancer defined by ERG or PTEN molecular subtypes. Cancer Epidemiol. Biomark. Prevent..

[30] Dickerman B.A., Ebot E.M., Healy B.C., Wilson K.M., Eliassen A.H., Ascherio A., Pernar C.H., Zeleznik O.A., Vander Heiden M.G., Clish C.B. (2020). A metabolomics analysis of adiposity and advanced prostate cancer risk in the health professionals follow-up study. Metabolites.

[31] Yang M., Ayuningtyas A., Kenfield S.A., Sesso H.D., Campos H., Ma J., Stampfer M.J., Chavarro J.E. (2016). Blood fatty acid patterns are associated with prostate cancer risk in a prospective nested case–control study. Cancer Cause. Control.

[32] Chavarro J.E., Kenfield S.A., Stampfer M.J., Loda M., Campos H., Sesso H.D., Ma J. (2013). Blood levels of saturated and monounsaturated fatty acids as markers of de novo lipogenesis and risk of prostate cancer. Am. J. Epidemiol..

[33] Huang J., Mondul A.M., Weinstein S.J., Derkach A., Moore S.C., Sampson J.N., Albanes D. (2019). Prospective serum metabolomic profiling of lethal prostate cancer. Int. J. Cancer.

[34] Huang J., Zhao B., Weinstein S.J., Albanes D., Mondul A.M. (2022). Metabolomic profile of prostate cancer-specific survival among 1812 Finnish Men. BMC Med..

[35] Östman J.R., Pinto R.C., Ebbels T.M.D., Thysell E., Hallmans G., Moazzami A.A. (2022). Identification of prediagnostic metabolites associated with prostate cancer risk by untargeted mass spectrometry-based metabolomics: a case-control study nested in the Northern Sweden Health and Disease Study. Int. J. Cancer.

[36] Röhnisch H.E., Kyrø C., Olsen A., Thysell E., Hallmans G., Moazzami A.A. (2020). Identification of metabolites associated with prostate cancer risk: a nested case-control study with long follow-up in the Northern Sweden Health and Disease Study. BMC Med..

[37] Breeur M., Ferrari P., Dossus L., Jenab M., Johansson M., Rinaldi S., Travis R.C., His M., Key T.J., Schmidt J.A. (2022). Pan-cancer analysis of pre-diagnostic blood metabolite concentrations in the European Prospective Investigation into Cancer and Nutrition. BMC Med..

[38] Kühn T., Floegel A., Sookthai D., Johnson T., Rolle-Kampczyk U., Otto W., von Bergen M., Boeing H., Kaaks R. (2016). Higher plasma levels of lysophosphatidylcholine 18:0 are related to a lower risk of common cancers in a prospective metabolomics study. BMC Med..

[39] Crowe F.L., Allen N.E., Appleby P.N., Overvad K., Aardestrup I.V., Johnsen N.F., Tjønneland A., Linseisen J., Kaaks R., Boeing H. (2008). Fatty acid composition of plasma phospholipids and risk of prostate cancer in a case-control analysis nested within the European Prospective Investigation into Cancer and Nutrition. Am. J. Clin. Nutrit..

[40] Dahm C.C., Gorst-Rasmussen A., Crowe F.L., Roswall N., Tjnøneland A., Drogan D., Boeing H., Teucher B., Kaaks R., Adarakis G. (2012). Fatty acid patterns and risk of prostate cancer in a case-control study nested within the European Prospective Investigation into Cancer and Nutrition. Am. J. Clin. Nutrit..

[41] Reichard C.A., Naelitz B.D., Wang Z., Jia X., Li J., Stampfer M.J., Klein E.A., Hazen S.L., Sharifi N. (2022). Gut microbiome-dependent metabolic pathways and risk of lethal prostate cancer: prospective analysis of a PLCO cancer screening trial cohort. Cancer Epidemiol. Biomark. Prevent..

[42] Koutros S., Meyer T.E., Fox S.D., Issaq H.J., Veenstra T.D., Huang W.Y., Yu K., Albanes D., Chu L.W., Andriole G. (2013). Prospective evaluation of serum sarcosine and risk of prostate cancer in the prostate, lung, colorectal and ovarian cancer screening trial. Carcinogenesis.

[43] Kurahashi N., Iwasaki M., Inoue M., Sasazuki S., Tsugane S. (2008). Plasma isoflavones and subsequent risk of prostate cancer in a nested case-control study: the Japan Public Health Center. J. Clin. Oncol..

[44] Lécuyer L., Victor Bala A., Demidem A., Rossary A., Bouchemal N., Triba M.N., Galan P., Hercberg S., Partula V., Srour B. (2021). NMR metabolomic profiles associated with long-term risk of prostate cancer. Metabolomics.

[45] Lin X., Lécuyer L., Liu X., Triba M.N., Deschasaux-Tanguy M., Demidem A., Liu Z., Palama T., Rossary A., Vasson M.P. (2021). Plasma metabolomics for discovery of early metabolic markers of prostate cancer based on ultra-high-performance liquid chromatography-high resolution mass spectrometry. Cancer. (Basel).

[46] Cernei N., Heger Z., Gumulec J., Zitka O., Masarik M., Babula P., Eckschlager T., Stiborova M., Kizek R., Adam V. (2013). Sarcosine as a potential prostate cancer biomarker–a review. Int. J. Mol. Sci..

[47] Kortesniemi M., Noerman S., Kårlund A., Raita J., Meuronen T., Koistinen V., Landberg R., Hanhineva K. (2023). Nutritional metabolomics: recent developments and future needs. Curr. Opin. Chem. Biol..

[48] Costello, L.C.; Franklin, R.B.; Narayan, P. Citrate in the Diagnosis of Prostate Cancer 1999. 10.1002/(sici)1097-0045(19990215)38:3<237::aid-pros8>3.0.co;2-o PMC4464828 10068348

[49] Lucarelli G., Loizzo D., Ferro M., Rutigliano M., Vartolomei M.D., Cantiello F., Buonerba C., Lorenzo G.D., Terracciano D., Cobelli O.D. (2019). Metabolomic profiling for the identification of novel diagnostic markers and therapeutic targets in prostate cancer: an update. Expert. Rev. Mol. Diagn..

[50] Ahmad F., Cherukuri M.K., Choyke P.L. (2021). Metabolic reprogramming in prostate cancer. Br. J. Cancer.

[51] Galey L., Olanrewaju A., Nabi H., Paquette J.-S., Pouliot F., Audet-Walsh É. (2024). Rediscovering citrate as a biomarker for prostate cancer. Nat. Rev. Urol..

[52] Pujana-Vaquerizo M., Bozal-Basterra L., Carracedo A. (2024). Metabolic adaptations in prostate cancer. Br. J. Cancer.

[53] Buszewska-Forajta M., Monedeiro F., Gołębiowski A., Adamczyk P., Buszewski B. (2022). Citric acid as a potential prostate cancer biomarker determined in various biological samples. Metabolites.

[54] Kumar D., Gupta A., Mandhani A., Sankhwar S.N. (2016). NMR spectroscopy of filtered serum of prostate cancer: a new frontier in metabolomics. Prostate.

[55] Kline E.E., Treat E.G., Averna T.A., Davis M.S., Smith A.Y., Sillerud L.O. (2006). Citrate concentrations in human seminal fluid and expressed prostatic fluid determined via ^1^ H nuclear magnetic resonance spectroscopy outperform prostate specific antigen in prostate cancer detection. J. Urol..

[56] Gregorio E.P., Alexandrino A.P., Schuquel I.T.A., da Costa W.F., Rodrigues M.A.d.F. (2019). Seminal citrate is superior to PSA for detecting clinically significant prostate cancer. Int. Braz. J. Urol..

[57] Andersen M.K., Høiem T.S., Claes B.S.R., Balluff B., Martin-Lorenzo M., Richardsen E., Krossa S., Bertilsson H., Heeren R.M.A., Rye M.B. (2021). Spatial differentiation of metabolism in prostate cancer tissue by MALDI-TOF MSI. Cancer Metab..

[58] Kumar D., Gupta A., Mandhani A., Sankhwar S.N. (2015). Metabolomics-derived prostate cancer biomarkers: fact or fiction?. J. Proteom. Res..

[59] Richard D.M., Dawes M.A., Mathias C.W., Acheson A., Hill-Kapturczak N., Dougherty D.M (2009). *L* -tryptophan: basic metabolic functions, behavioral research and therapeutic indications. Int. J. Tryptoph. Res..

[60] Badawy A.A.-B. (2017). Kynurenine pathway of tryptophan metabolism: regulatory and functional aspects. Int. J. Tryptoph. Res..

[61] Chen Y., Xie Y., Ci H., Cheng Z., Kuang Y., Li S., Wang G., Qi Y., Tang J., Liu D. (2024). Plasma metabolites and risk of seven cancers: a two-sample mendelian randomization study among European descendants. BMC Med..

[62] Liu X., Zheng Y., Guasch-Ferré M., Ruiz-Canela M., Toledo E., Clish C., Liang L., Razquin C., Corella D., Estruch R. (2019). High plasma glutamate and low glutamine-to-glutamate ratio are associated with type 2 diabetes: case-cohort study within the PREDIMED trial. Nutrit. Metabol. Cardiovascul. Dis..

[63] Koochekpour S., Majumdar S., Azabdaftari G., Attwood K., Scioneaux R., Subramani D., Manhardt C., Lorusso G.D., Willard S.S., Thompson H. (2012). Serum glutamate levels correlate with gleason score and glutamate blockade decreases proliferation, migration, and invasion and induces apoptosis in prostate cancer cells. Clin. Cancer Res..

[64] Heger Z., Gumulec J., Cernei N., Polanska H., Raudenska M., Masarik M., Eckschlager T., Stiborova M., Adam V., Kizek R. (2016). Relation of exposure to amino acids involved in sarcosine metabolic pathway on behavior of non-tumor and malignant prostatic cell lines. Prostate.

[65] Lin Y., Yang Z., Li J., Sun Y., Zhang X., Qu Z., Luo Y., Zhang L. (2022). Effects of glutamate and aspartate on prostate cancer and breast cancer: a Mendelian randomization study. BMC Genom..

[66] Debras C., Chazelas E., Srour B., Druesne-Pecollo N., Esseddik Y., Szabo de Edelenyi F., Agaësse C., De Sa A., Lutchia R., Gigandet S. (2022). Artificial sweeteners and cancer risk: results from the NutriNet-Santé population-based cohort study. PLoS Med.

[67] Grimm C., Hofstetter G., Aust S., Mutz-Dehbalaie I., Bruch M., Heinze G., Rahhal-Schupp J., Reinthaller A., Concin N., Polterauer S. (2013). Association of gamma-glutamyltransferase with severity of disease at diagnosis and prognosis of ovarian cancer. Br. J. Cancer.

[68] Yang J., Bai W., Zeng X., Cui C. (2019). Gamma glutamyl peptides: the food source, enzymatic synthesis, kokumi-active and the potential functional properties – a review. Trend. Food Sci. Technol..

[69] Jentzmik F., Stephan C., Miller K., Schrader M., Erbersdobler A., Kristiansen G., Lein M., Jung K. (2010). Sarcosine in urine after digital rectal examination fails as a marker in prostate cancer detection and identification of aggressive tumours. Eur. Urol..

[70] Jentzmik F., Stephan C., Lein M., Miller K., Kamlage B., Bethan B., Kristiansen G., Jung K. (2011). Sarcosine in prostate cancer tissue is not a differential metabolite for prostate cancer aggressiveness and biochemical progression. J. Urol..

[71] Johansson M., Guelpen B.V., Vollset S.E., Hultdin J., Bergh A., Key T., Midttun Ø., Hallmans G., Ueland P.M., Stattin P (2009). One-carbon metabolism and prostate cancer risk: prospective investigation of seven circulating B vitamins and metabolites. Cancer Epidemiol., Biomark. Prevent..

[72] Chan J.M., Gann P.H., Giovannucci E.L. (2005). Role of diet in prostate cancer development and progression. J. Clin. Oncol..

[73] Dagnelie P.C., Schuurman A.G., Goldbohm R.A., Van Den Brandt P.A. (2004). Diet, anthropometric measures and prostate cancer risk: a review of prospective cohort and intervention studies. BJU Int..

[74] Sun S., Li X., Ren A., Du M., Du H., Shu Y., Zhu L., Wang W. (2016). Choline and betaine consumption lowers cancer risk: a meta-analysis of epidemiologic studies. Sci. Rep..

[75] Zhou X., Mao J., Ai J., Deng Y., Roth M.R., Pound C., Henegar J., Welti R., Bigler S.A. (2012). Identification of plasma lipid biomarkers for prostate cancer by lipidomics and bioinformatics. PLoS One.

[76] Scheinberg T., Mak B., Butler L., Selth L., Horvath L.G. (2023). Targeting lipid metabolism in metastatic prostate cancer. Ther. Adv. Med. Oncol..

[77] Zang X., Jones C.M., Long T.Q., Monge M.E., Zhou M., Walker L.D., Mezencev R., Gray A., McDonald J.F., Fernández F.M. (2014). Feasibility of detecting prostate cancer by ultraperformance liquid chromatography–mass spectrometry serum metabolomics. J. Proteom. Res..

[78] Lokhov P.G., Dashtiev M.I., Moshkovskii S.A., Archakov A.I. (2010). Metabolite profiling of blood plasma of patients with prostate cancer. Metabolomics.

[79] Adams C.D., Richmond R., Ferreira D.L.S., Spiller W., Tan V., Zheng J., Würtz P., Donovan J., Hamdy F., Neal D. (2019). Circulating metabolic biomarkers of screen-detected prostate cancer in the ProtecT study. Cancer Epidemiol. Biomark. Prevent..

[80] Koralek D.O., Peters U., Andriole G., Reding D., Kirsh V., Subar A., Schatzkin A., Hayes R., Leitzmann M.F. (2006). A prospective study of dietary alpha-linolenic acid and the risk of prostate cancer (United States). Cancer Cause. Control.

[81] Crowe F.L., Key T.J., Appleby P.N., Travis R.C., Overvad K., Jakobsen M.U., Johnsen N.F., Tjønneland A., Linseisen J., Rohrmann S. (2008). Dietary fat intake and risk of prostate cancer in the European Prospective Investigation into Cancer and Nutrition. Am. J. Clin. Nutr..

[82] Korbecki J., Bajdak-Rusinek K. (2019). The effect of palmitic acid on inflammatory response in macrophages: an overview of molecular mechanisms. Inflamm. Res..

[83] Dai X., Wang H., Zhong R., Li J., Hou Y. (2024). Causality of genetically determined metabolites on susceptibility to prevalent urological cancers: a two-sample mendelian randomization study and meta-analysis. Front. Genet..

[84] Chen Y., Xie Y., Ci H., Cheng Z., Kuang Y., Li S., Wang G., Qi Y., Tang J., Liu D. (2024). Plasma metabolites and risk of seven cancers: a two-sample mendelian randomization study among European Descendants. BMC Med..

[85] Xu B., Chen Y., Chen X., Gan L., Zhang Y., Feng J., Yu L. (2021). Metabolomics profiling discriminates prostate cancer from benign prostatic hyperplasia within the prostate-specific Antigen gray zone. Front. Oncol..

