# Supplementary material for: Prostate cancer risk biomarkers from large cohort and prospective metabolomics studies: A systematic review
Source: Transl Oncol. 2024 Nov 23;51:102196. doi: 10.1016/j.tranon.2024.102196 (PMC11625367; doi:10.1016/j.tranon.2024.102196)
Supplement: Supplementary file 1 [file mmc1.docx]

Supplementary Table 1. Systematic reviews studying metabolomics and Prostate cancer biomarkers.

| **Criteria** | **"Prostate Cancer Risk Biomarkers from Large Cohort and Prospective Metabolomics Studies"** | **Bansal et al., 2022** | **Kdadra et al., 2019** |
| --- | --- | --- | --- |
| **Main Goal** | To identify pre-diagnostic or risk assessment prostate cancer biomarkers in serum/plasma from patients recruited in prospective large cohort studies | To provide an up-to-date summary of chronologically existing metabolomics PC biomarkers, their potential to improve clinical PC diagnosis and to reduce the proliferation and monitoring of PC. | To evaluate various metabolomics biomarkers and their clinical utility in prostate cancer diagnosis, prognosis, progression, aggressiveness, recurrence, and treatment response. |
| **Search Strategy** | A search was performed within publicly available repositories of large cohort studies; then, articles involving prostate cancer metabolomics biomarkers investigation in serum/plasma for each large cohort study were retrieved from PUBMED between October-November 2023. | Systematic search using PubMed, with search terms focused on prostate cancer metabolomics. January 2004-October 2021 | Comprehensive search, with specific inclusion of human prostate cancer metabolomics studies. July 2008-July 2018 |
| **Selection Criteria** | Prospective/longitudinal/large cohort metabolomics studies that used serum/plasma samples collected at baseline, with a PCa diagnosis made within at least one year after sample collection. 29 large cohort studies were included. | Human PC serum/plasma metabolite biomarkers for identification, progression  or reoccurrence and their clinical utility using metabolomics analytical platforms. 27 blood-based metabolomic studies, with only eight being for PCa risk assessment in large cohort studies | Focused on human metabolomics biomarkers, excluding animal and cell-based studies. 59 studies analyzing blood, urine, tissue, seminal fluids. 17 blood-based metabolomic studies, of which only six were nested case-control studies for PCa risk |
| **Adherence to PRISMA Guidelines** | Yes, PRISMA-compliant, with a flowchart showing study selection | Yes, followed PRISMA guidelines and provided detailed criteria for inclusion/exclusion | Yes, adheres to PRISMA with a systematic approach and flowchart for article selection |
| **Strength of Findings** | A list of metabolites (mentioned across large cohort studies) was provided and suggested for validation in future studies | Highlights different types of biomarkers identified in serum/plasma. | Provides a comprehensive review of metabolomics biomarkers and evaluates their utility across studies |
| **Challenges** | Different metabolomics platforms and methods between studies create variability in findings | Variability in technical methods and statistical approaches, and need for more standardized methodologies | Need for validation of biomarkers and harmonization of analytical techniques between studies |
| **Advantages and limitations** | Large sample sizes and prospective study designs increase the validity of findings. Since some studies reported conflicting results, it is only possible to discuss potential mechanism of actions. Lack of enough data to perform metanalysis for the coincident metabolites. | Emphasizes clinical relevance and the potential of biomarkers for diagnostic use. Limited sample sizes and lack of standardization in study methods affect generalizability | Provides a thorough review of existing literature and discusses both diagnostic and prognostic biomarkers. Many studies lack validation, and biomarker reproducibility remains a challenge |
