# Supplementary material for: Prostate cancer risk biomarkers from large cohort and prospective metabolomics studies: A systematic review
Source: Transl Oncol. 2024 Nov 23;51:102196. doi: 10.1016/j.tranon.2024.102196 (PMC11625367; doi:10.1016/j.tranon.2024.102196)
Supplement: Supplementary file 2 [file mmc2.docx]

Supplementary Table 2. Description of Prospective Cohorts screened in this study

| **COHORT STUDY** | **WEBSITE** | **COUNTRY** | **ENRROLLMENT START DATE** | **STATUS** | **PARTICIPANTS** | **NUMBER OF PROSTATE CANCER (JAN/2023)** |
| --- | --- | --- | --- | --- | --- | --- |
| Janus Serum Bank  (JANUS) | http://www.kreftregisteret.no/en/Research/Janus-Serum-Bank/ | Norway | 1972 | Ongoing | 318,628 | 16,941 |
| Clue Cohort Study- Clue I: Campaign Against Cancer and Stroke  (Clue I) | https://publichealth.jhu.edu/george-w-comstock-center-for-public-health-research-and-prevention/research-activities/the-clue-studies | US | 1974 | Ongoing | 26,144 | 775 |
| New York State Cohort  (NYSC) | - | US | 1980 | Finished | 57,968 | - |
| Cancer Prevention Study II Nutrition Cohort  (CPS-II Nutrition) | <https://www.cancer.org/research/population-science/cancer-prevention-and-survivorship-research-team/acs-cancer-prevention-studies/cancer-prevention-study-2.html> | US | 1982 | Ongoing | 184,000 | 10, 467 |
| Physicians Health Study I and II  (PHS I/PHS II) | <http://phs.bwh.harvard.edu/> | US | 1982 | Finished | 29,071 | 3,562 |
| Alpha-Tocopherol Beta-Carotene Cancer Prevention Study  (ATBC) | <http://atbcstudy.cancer.gov/> | Finland | 1985 | Finished | 29,133 | 2,725 |
| Carotene and Retinol Efficacy Trial (CARET) | http://www.compass.fhcrc.org/caretWeb | US | 1985 | Finished | 18,314 | 1,256 |
| Health Professionals Follow-up Study  (HPFS) | <http://www.hsph.harvard.edu/hpfs/> | US | 1986 | Ongoing | 51,529 | 9,209 |
| Netherlands Cohort Study  (NLCS) | https://epi.grants.cancer.gov/cohort-consortium/members/nlcs.html | The Netherlands | 1986 | Ongoing | 120,852 | - |
| Northern Sweden Health and Disease Study  (NSHDS) | <https://www.umu.se/enheten-for-biobanksforskning/provsamlingar-och-register/northern-sweden-health-and-disease-study/> | Sweden | 1986 | Ongoing | 143,629 | 4,745 |
| Shanghai Cohort Study  (SCS) | https://www.schs.pitt.edu/methods/shanghai-cohort-study/ | China | 1986 | Ongoing | 18,244 | 366 |
| Atherosclerosis Risk in Communities  (ARIC) | https://www2.cscc.unc.edu/aric/desc | US | 1987 | Ongoing | 15,792 | 887 |
| Clue Cohort Study- Clue II  (CLUE II) | <https://publichealth.jhu.edu/george-w-comstock-center-for-public-health-research-and-prevention/research-activities/the-clue-studies> | US | 1989 | Finished | 32,894 | 842 |
| Melbourne Collaborative Cohort Study  (MCCS) | http://www.pedigree.org.au/pedigree-studies/health2020.aspx | Australia | 1990 | Ongoing | 41,513 | 2,516 |
| European Prospective Investigation into Cancer and Nutrition (EPIC) | <http://epic.iarc.fr/> | 10 European countries | 1992 | Ongoing | 521,323 | 7,572 |
| Agricultural Health Study  (AHS) | http://aghealth.nih.gov/ | US | 1993 | Finished | 83,028 | 3,326 |
| Prostate Cancer Prevention Trial  (PCPT) | https://www.swog.org/clinical-trials/biospecimen-resources/pcpt-biorepository | US | 1993 | Finished | 18,880 | 2,484 |
| Prostate, Lung, Colon, and Ovarian Cancer Study  (PLCO) | <https://biometry.nci.nih.gov/cdas/plco/> | US | 1993 | Ongoing | 155,000 | 8,470 |
| Singapore Chinese Health Study  (SCHS) | https://sph.nus.edu.sg/research/cohort-schs/ | China | 1993 | Ongoing | 63,257 | 694 |
| The SUpplementation en VItamines et Minéraux AntioXydants Study  (SU.VI.MAX) | <http://exposome-explorer.iarc.fr/cohorts/38> | France | 1994 | Finished | 12,735 | 171 |
| Multiethnic Cohort Study  (MEC) | https://www.uhcancercenter.org/for-researchers/mec-cohort-composition | US | 1995 | Ongoing | 215,251 | 10,026 |
| NIH-AARP Diet and Health Study  (NIH-AARP) | <https://dceg.cancer.gov/research/who-we-study/nih-aarp-diet-health-study> | US | 1995 | Finished | 567,000 | 32,015 |
| Cohort of Swedish Men  (COSM) | https://www.simpler4health.se/?languageId=1 | Sweden | 1997 | Ongoing | 50,000 | 6,210 |
| Health, Aging and Body Composition  (HEALTH ABC) | https://healthabc.nia.nih.gov | US | 1997 | Finished | 3,075 | - |
| Swedish National March Cohort  (SNMC) | http://ki.se/en/meb/the-swedish-national-march-cohort-nmc | Sweeden | 1997 | Finished | 43,804 | 1,257 |
| The Colon Cancer Family RegistryCohort  (CCFR) | https://coloncfr.org | US, Canada, Australia, New Zealand | 1998 | Ongoing | 37,333 | 722 |
| VITamins And Lifestyle  (VITAL) | https://www.fredhutch.org/en/labs/phs/projects/cancer-prevention/projects/vital.html | US | 2000 | Ongoing | 77,738 | 2,590 |
| Adventist Health Study-2  (AHS-2) | https://adventisthealthstudy.org | US and Canada | 2001 | - | 96,000 | - |
| Mano A Mano, the Mexican American Cohort  (MAC) | [http://www.mano-mano.us](http://www.mano-mano.us/) | US | 2001 | Ongoing | 25,466 | 128 |
| Southern Community Cohort Study  (SCCS) | http://www.southerncommunitystudy.org/ | US | 2001 | Ongoing | 85,401 | 1,691 |
| Selenium and Vitamin E Cancer Prevention Trial  (SELECT) | https://www.swog.org/clinical-trials/biospecimen-resources/select-biorepository | US | 2001 | Finished | 34,897 | 2,593 |
| The Shanghai Men’s Health Study  (SMHS) | http://www.mc.vanderbilt.edu/swhs-smhs | China | 2002 | Ongoing | 61,582 | 551 |
| Generation Scotland: Scottish Family Health Study  (GSSFHS) | https://www.ed.ac.uk/generation-scotland | Scotland | 2003 | Ongoing | 24,066 | 203 |
| The Canadian Partnership for Tomorrow Project  (CPTP) | http://www.partnershipfortomorrow.ca/ | Canada | 2008 | Ongoing | 331,359 | 2,661 |
| Polish - Norwegian Study  (PONS) | www.projectpons.pl (not operating at the moment) | Poland | 2010 | Ongoing | 13,147 | 62 |
| The Japan Public Health Center-Based Study Cohort (JPHC) | <https://epi.ncc.go.jp/en/jphc/> | Japan | 2011 | Ongoing | 140,000 | 1,386 |
| Connect for Cancer Prevention Study  (CONNECT) | https://dceg.cancer.gov/research/who-we-study/cohorts/connect | US | 2021 | Ongoing | 200,000 | - |
|  |  |  |  | TOTAL | 3,748,152 | 128,636 |
