# Supplementary material for: Prostate cancer risk biomarkers from large cohort and prospective metabolomics studies: A systematic review
Source: Transl Oncol. 2024 Nov 23;51:102196. doi: 10.1016/j.tranon.2024.102196 (PMC11625367; doi:10.1016/j.tranon.2024.102196)
Supplement: Supplementary file 3 [file mmc3.docx]

**Supplementary Table 3:** Quality assessment based on *Cochrane Handbook for Systematic Reviews of Interventions*

| **Article** | **Bias Due to Confounding (Domain 1)** | **Bias in Selection of Participants (Domain 2)** | **Bias in Classification of Exposure (Domain 3)** | **Bias Due to Deviations from Intended Interventions (Domain 4)** | **Bias in Measurement of Outcomes (Domain 5)** | **Bias in Selection of Reported Results (Domain 6)** |
| --- | --- | --- | --- | --- | --- | --- |
| De Vogel *et al*. [17] | Low | Some concerns | Low | Low | Some concerns | Some concerns |
| Koutros *et al.* [25] | Low | Low | Low | Some concerns | Low | Low |
| Harvei *et al.*[26] | Low | Some concerns | Low | Low | Some concerns | Some concerns |
| Huang *et al*. [27] | Some concerns | Some concerns | Low | Some concerns | Some concerns | Some concerns |
| Wang *et al.* [28] | Low | Some concerns | Low | Some concerns | Low | Low |
| Feng *et al.* [29] | Some concerns | Some concerns | Low | Low | Some concerns | Some concerns |
| Dickerman *et al.* [30] | Low | Low | Low | Some concerns | Low | Low |
| Yang *et al.* [31] | Low | Low | Low | Low | Low | Low |
| Chavarro *et al.* [32] | Low | Low | Low | Low | Low | Some concerns |
| Mondul *et al.* [16] | Low | Low | Low | Low | Low | Some concerns |
| Mondul *et al.* [15] | Low | Some concerns | Low | Low | Low | Some concerns |
| Huang *et al*. [17] | \| Low \| \| --- \| | Low | Low | Some concerns | Some concerns | Some concerns |
| Huang *et al.* [33] | Low | Low | Low | Some concerns | Some concerns | Some concerns |
| Huang *et al*. [22] | Low | Low | Low | Some concerns | Low | Some concerns |
| Huang *et al*. [34] | Low | Low | Low | Some concerns | Low | Some concerns |
| Östman *et al*. [35] | Some concerns | Some concerns | Low | Some concerns | Low | Some concerns |
| Röhnisch *et al.* [36] | Low | Low | Low | Some concerns | Low | Some concerns |
| Breeur *et al.* [37] | Low | Low | Low | Low | Low | Low |
| Schmidt *et al*. [21] | Low | Low | Low | Low | Low | Some concerns |
| Schmidt *et al*. [19] | Low | Low | Low | Low | Low | Low |
| Kuhn *et al*. [38] | Low | Some concerns | Some concerns | Low | Some concerns | Low |
| Crowe *et al.* [39] | Low | Low | Low | Some concerns | Some concerns | Some concerns |
| Dahm *et al.* [40] | Low | Low | Low | Low | Some concerns | Some concerns |
| Reichard *et al*. [41] | Low | Some concerns | Some concerns | Low | Low | Some concerns |
| Huang *et al*. [18] | Low | Low | Low | Low | Low | Some concerns |
| Koutros *et al*. [42] | Low | Low | Low | Some concerns | Low | Some concerns |
| Kurahashi *et al*. [43] | Low | Low | Low | Low | Low | Low |
| Lécuyer *et al*. [44] | Low | Low | Low | Some concerns | Low | Some concerns |
| Lin *et al.* [45] | Low | Low | Low | Some concerns | Low | Some concerns |
