# Supplementary material for: Prostate cancer risk biomarkers from large cohort and prospective metabolomics studies: A systematic review
Source: Transl Oncol. 2024 Nov 23;51:102196. doi: 10.1016/j.tranon.2024.102196 (PMC11625367; doi:10.1016/j.tranon.2024.102196)
Supplement: Supplementary file 4 [file mmc4.docx]

Supplementary Table 4: Adjusted covariates in each prospective study

| **Author** | **Cohort** | **Confounding factors** | **Adjusted covariates in the model** | **Relevance of the association** |
| --- | --- | --- | --- | --- |
| De Vogel et al | JANUS | Smoking behavior, physical activity, body mass index (BMI)  and educational level | Highest education, smoking habits, physical activity and BMI. Serum creatinine  Stratification by folate status and MTHFR 677 T | Higher education and BMI bellow 25 was more common among cases. |
| Koutros et al | JANUS | BMI and smoking habits | BMI and smoking | Negligible effect on the observed organochlorine-disease associations |
| Harvei *et al.*[26] | JANUS | Stage, age, lag time, histological differentiation | Stage, age, lag time, histological differentiation | Clear biological explanations are lacking |
| Huang *et al*. [27] | Clue | Age, race, education, smoking, hours since last meal, supplement use | Total lipid levels, hors since last meal, education | In CLUE I, the odds ratio estimates for α -  tocopherol were slightly attenuated for the highest fifth after adjustment  for serum total lipids; in the CLUE II study, results  were unchanged after adjustment. The association between ascorbic acid and prostate cancer did not change after adjustment  for hours since last meal and years of education in the CLUE II study. |
| Wang *et al.* [28] | CPS-II | Race, family history of PCa, PSA testing, BMI, physical activity, smoking status, time since the last meal | Race, family history of PCa, PSA testing, BMI, physical activity, smoking status, time since the last meal | No significant |
| Feng *et al.* [29] | PHS/HPFS | Age, PSA testing, season and calendar year of blood draw | Age, cohort, height, BMI, physical activity, smoking status, fasting status, and season | ND |
| Dickerman *et al.* [30] | PHS/HPFS | Age, BMI, Race, smoking status, T2DM, family history of PCa, PSA testing | Adiposity measures | BMI, waist circumference, and derived fat mass were associated with metabolic alterations but not with risk of advanced PCa. |
| Yang *et al.* [31] | PHS/HPFS | Age, smoking status | Age, smoking status | No significant |
| Chavarro *et al.* [32] | PHS/HPFS | Age, smoking status | Height, BMI | No significant |
| Mondul *et al.* [16] | ATBC | General risk factors, smoking, medical history, BMI, smoking, education, intakes of total energy, fruit, vegetables, red meat, alcohol, caffeine, supplements | Age, batch, trial supplement assignment, first-degree family history of PCa, physical activity | No significant |
| Mondul *et al.* [15] | ATBC | Trial treatment group, family history of PCa, BPH, physical activity, BMI, smoking, cholesterol, retinol, alpha tocopherol | Age and date of blood collection | No significant |
| Huang *et al*. [17] | ATBC | Risk factors | Trial intervention group, family history of PCa, BPH, BMI, physical activity, cigarettes, PSA, HDL, cholesterol, retinol, alpha tocopherol | No significant |
| Huang *et al.* [33] | ATBC | Lifestyle factors | BMI, cigarettes, cholesterol, alpha-tocopherol, retinol, fasting hours, ATBC intervention group | No significant |
| Huang *et al*. [22] | ATBC | Risk factor questionnaires | Time from blood collection, BMI, cigarettes, cholesterol, hypertension, T2DM | No significant |
| Huang *et al*. [34] | ATBC | Lifestyle risk factors, height, weight, heart rate, blood pressure | BMI, smoking, cholesterol, calendar years of cancer diagnosis | No significant |
| Östman *et al*. [35] | NSHDS | Age, BMI, SBP, DBP, cholesterol, stage of cancer | Age, disease aggressiveness | No significant |
| Röhnisch *et al.* [36] | NSHDS | Height, weight, blood pressure, health examination | BMI, age, sample storage time | Glycerophospholipids strongly associated in older subjects |
| Breeur *et al.* [37] | EPIC | Health risk factors | Cancer risk factors, BMI, | No significant |
| Schmidt *et al*. [21] |  | Age at blood collection, baseline values of body  mass index, smoking, alcohol intake, educational and marital level | Age at blood collection, baseline values of body  mass index, smoking, alcohol intake, educational and marital level | No significant |
| Schmidt *et al*. [19] | EPIC | Study center, length of follow-up, age, time of day, fasting status | Age at blood collection, BMI, study center, smoking, alcohol, education, marital status | Time to diagnosis associated with some metabolites |
| Kuhn *et al*. [38] | EPIC | Habitual diet, smoking, alcohol, physical activity, socio-economic status, anthropometric measures | Age, smoking, alcohol, physical activity, waist circumference, BMI, education | No significant |
| Crowe *et al.* [39] | EPIC | Age, BMI, smoking, alcohol, physical activity, education | BMI, smoking, alcohol intake, educational level, marital status, physical activity | Palmitic acid (risk increased after adjustment) |
| Dahm *et al.* [40] | EPIC | Study center, age at enrollment, time from last meal | BMI, smoking, alcohol, education, physical activity | No significant |
| Reichard *et al*. [41] | PLCO | Race, age, time of blood draw, enrollment date | BMI, PSA | Adjustment increase risk for choline and betaine levels |
| Huang *et al*. [18] | PLCO | Height, weight, smoking, family history of cancer, physical activity, medications | Age, race, study center, study year, date of blood collection | NO SIGNIFICANT |
| Koutros *et al*. [42] | PLCO | Age, study center, family history of PCa, smoking, BMI, diabetes, years of follow-up, marital status, hypertension, PSA, supplements | Age, study center, family history of PCa, diabetes, smoking | BMI and age: no significant |
| Kurahashi *et al*. [43] | JPHC | Lifestyle factors, smoking, alcohol | Smoking, alcohol, marital status, green tea intake, intake of protein, fiber, vegetables and dairy food | Localized cases: genistein association is significant after adjustment |
| Lécuyer *et al*. [44] | SU.VI.MAX | Age, BMI, Smoking, Alcohol, Physical Activity | Age, BMI, Smoking, Alcohol, Physical Activity, educational leve, family history of PCa | No significant |
| Lin *et al.* [45] | SU.VI.MAX | Clinical factors | Clinical factors | Some metabolites significant after adjustment |
